# Supramolecular Complexes of β-Cyclodextrin with Clomipramine and Doxepin: Effect of the Ring Substituent and Component of Drugs on Their Inclusion Topologies and Structural Flexibilities

**DOI:** 10.3390/ph13100278

**Published:** 2020-09-29

**Authors:** Thammarat Aree

**Affiliations:** Department of Chemistry, Faculty of Science, Chulalongkorn University, Bangkok 10330, Thailand; athammar@chula.ac.th; Tel.: +66-2-2187584; Fax: +66-2-2187598

**Keywords:** β-cyclodextrin, clomipramine, doxepin, tricyclic antidepressants, X-ray analysis, density functional theory (DFT) calculation

## Abstract

Depression is a global threat. Tricyclic antidepressants (TCAs) are still efficacious in treating depression, albeit with more side effects. Cyclodextrins (CDs) with a suitable nanocavity are potential drug carriers and can enhance the drug bioavailability. Aiming for an atomistic understanding of the CD encapsulation facilitating the improvement of drug stability and the reduction of side effects, a comprehensive study series of the β-CD–TCA inclusion complexes through single crystal X-ray diffraction and density functional theory (DFT) calculation was undertaken. This work reports the supramolecular complexes of β-CD with two pivotal TCAs, clomipramine (CPM; **1**) and doxepin (DXP; **2**). The different inclusion topologies of the β-CD–TCA complexes were notable. X-ray analysis revealed that, in **1,** the CPM B-ring (without chloro group) was entrapped in the β-CD cavity, whereas, in **2,** the *E*-DXP A-ring and the *Z*-DXP B-ring were disordered in the cavity, yielding energetically favorable complexes primarily maintained by intermolecular C–H**⋯**π interactions, as indicated by DFT calculation. Because both wings of TCAs were similar, an alternative inclusion scenario of the A-ring was evidenced crystallographically in four other TCA complexes. The enhanced TCA thermodynamic stabilities via CD inclusion complexation helped to reduce the side effects and to increase the bioavailability. Moreover, the scrutinization of six TCAs in different lattice circumstances revealed the greater TCA structural flexibilities for their optimum pharmacological activity while binding with proteins.

## 1. Introduction

**Scheme 1:**
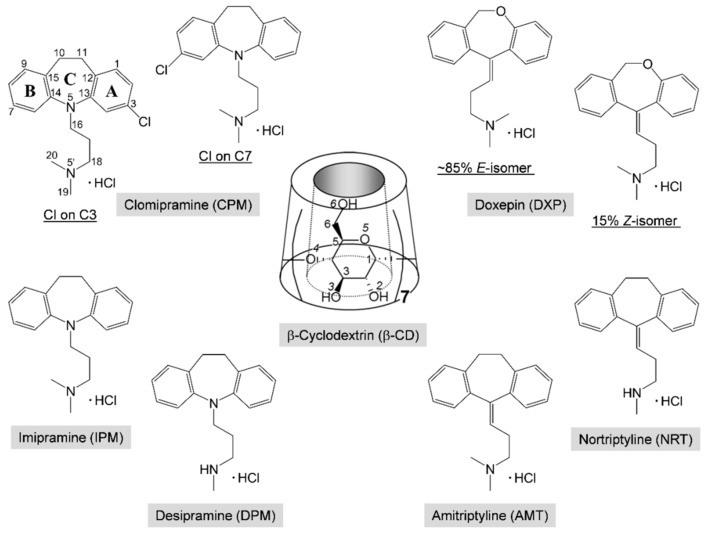
Chemical structures and atom numbering of tricyclic antidepressants (TCAs), clomipramine (CPM), doxepin (DXP), imipramine (IPM), desipramine (DPM), amitriptyline (AMT), nortriptyline (NRT), and β-cyclodextrin (CD). Note the two positional isomers of CPM with Cl on C3 of the A-ring (3-chloro-IPM) and on C7 of the B-ring (7-chloro-IPM), and the *E*-(*trans*) and *Z*-(*cis*) isomers of DXP.

Depression is a common and serious mental illness. About 300 million people of all ages in the world have depression according to WHO findings [1]. Clomipramine (CPM; Anafranil) and doxepin (DXP; Silenor) share structural similarities with the first-generation tricyclic antidepressant (TCA) drugs, Scheme 1. CPM is a chloro derivative of imipramine (IPM; Tofranil), which gives an active metabolite desipramine (DPM; Norpramin) via demethylation in the body (liver). DXP is resulted from the substitution of oxygen for the carbon of amitriptyline (AMT; Elavil), which undergoes metabolism to give nortriptyline (NRT; Pamelor). TCAs have still been widely used due to their cheap prices and high clinical efficacy, though they have more side effects [2]. However, the second-generation antidepressants, selective serotonin reuptake inhibitors (SSRIs) with equivalent efficacy to that of TCAs, are relatively safe and have fewer side effects, though they have higher costs.

TCAs comprise a (6-7-6)-tricyclic core (viz., the A-C-B-rings, respectively) connected at N5/C5 with a three-carbon-length alkylamine side chain, giving an overall shape of the butterfly (Scheme 1). The TCA molecules are rather flexible because they occupy a large potential energy surface with many energetically favorable conformations, as theoretically investigated by molecular mechanics and semiempirical Austin model 1 (AM1) calculations [3]. The secondary amine TCAs (e.g., NRT, DPM) are selective inhibitors of norepinephrine, and the tertiary amine TCAs (e.g., AMT, IPM, CPM, DXP) block the reuptake of both serotonin and norepinephrine [4]. Among TCAs, CPM and IPM have high affinity with serotonin, pharmacologically similar to SSRIs [5]. Substitution of chloro group at C3/C7 position on the aromatic A/B-ring of IPM yields CPM with the highest efficacy in the depressive disorder treatment [6]. DXP is marketed as a mixture of ~85% *E*-(*trans*-)-isomer and 15% *Z*-(*cis*-)-isomer; *Z*-form is considered to have greater antidepressive effects [7]. DXP in a relatively high starting dose can be used the next day after stopping administration of CPM without a washed out period, inferring that the two drugs have insignificant interactions [8].

Cyclic oligosaccharides comprising 6, 7, and 8 d-glucose units are well known as α-, β-, and γ-CDs, respectively. CDs resemble a hollow, truncated cone and have amphiphilic properties with hydrophobic central cavity and hydrophilic rims (Scheme 1). Hence, they can accommodate a number of guest molecules fitting to their nanocavity, yielding inclusion complexes, which have the potential to be applied in various industries, e.g., agriculture, food, and cosmetics [9]. In pharmaceutical technology, CDs are used for improving solubility, stability, and bioavailability of drugs [10,11,12]. The side effects of TCAs can be reduced via the CD encapsulation [10]. Extensive research on CD inclusion complexes with TCAs has been carried out, mostly for β-CD in solution at 298 K, over the past 30 years (Table S1, Supplementary Materials). CDs prefer harboring the TCA side chain to the aromatic A-/B-rings, forming moderately stable equimolar inclusion complexes in gas and solution phases. By contrast, in the solid state, the inclusion mode of the aromatic A-ring has exclusively been evidenced thus far [13,14,15].

For the CD–TCA inclusion complexes, whereas the structurally related TCA drugs NRT, AMT, DPM, and IPM receive greater attention (Table S1), the isomeric drugs CPM and DXP remain to be explored. CPM in complex with CDs is the least studied among TCAs. CPM is found to form stable equimolar inclusion complexes with β-CD and hydroxypropyl-β-CD (HP-β-CD) with binding constants of 9.42 × 10^3^ and 9.58 × 10^3^ M^−1^ based on UV-vis data [16]. The aromatic A-ring bearing chloro group is enclosed in the CD cavity as indicated by NMR data [16], Table S1. The stable 1:1 inclusion complexes of β-CD with DXP, IPM, NRT, and AMT have association constants in the range of 8.70–23.90 × 10^3^ M^−1^, whereas the corresponding α-CD complexes are relatively weak, 0.05–0.09 × 10^3^ M^−1^, as investigated using ion-selective electrodes [17], Table S1. NMR data reveal that the aromatic A-ring of DXP is found deeper than the B-ring in the β-CD cavity [18]. However, UV-vis and fluorescence spectroscopies as well as molecular modeling indicate the contrary for β-CD complexes with DPM, IPM, and AMT, i.e., the TCA side chain is merely included in the CD cavity [17,19].

The CD encapsulation of DXP receives more attention. The *E*-isomer of DXP forms a more stable inclusion complex with β-CD than does the *Z*-isomer, as indicated by respective binding constants of 3.60 × 10^4^ and 2.27 × 10^4^ M^−1^ from capillary electrophoresis [20]. Semiempirical PM3 calculations show that the equimolar β-CD–IPM and β-CD–DXP inclusion complexes are thermodynamically stable; the aromatic moiety is partly included in the CD cavity [21], Table S1. Moreover, in solution, DXP forms 1:2 inclusion complexes with α- and β-CDs such that the two aromatic A- and B-rings are embedded in the CD cavities with binding constants of 14.7–16.5 × 10^3^ and 16.2–19.6 × 10^3^ M^−1^, as deduced from UV-vis and fluorescence data, respectively [22]. By contrast, in the gas phase, PM3 calculations suggest the energetically favorable 1:1 α-CD–DXP inclusion complex with the side chain enclosed in the cavity and maintained in position by van der Waals forces and hydrophobic interactions [22]. The weak host–guest interactions are confirmed in solution for the 3:1 β-CD–DXP inclusion complex based on UV-vis and fluorescence data [23]. The binding constants are estimated for the 1:1 β-CD–DXP complex with the inclusion of the aromatic moiety, 397 M^−1^ (UV-vis) and 624 M^−1^ (fluorescence) [23].

Recently, crystallographic evidences for the β-CD encapsulation of four important TCAs, AMT base [13], AMT HCl [14], NRT HCl [14], and DPM HCl and IPM HCl [15], have been disclosed unequivocally. Because the four drugs are structurally related, these stable complexes have an exclusive inclusion mode with the TCA aromatic A-ring moiety embedded in the β-CD cavity and directed to the O6-side, establishing host–guest C/O–H**⋯**π interactions. Moreover, DFT calculations reveal that, in vacuum, the bimodal β-CD–TCA inclusion complexes with both the A-ring and the side chain portions competitively bound to the β-CD cavity are energetically favorable.

The thorough literature review raises two hypotheses for the β-CD encapsulation of isomeric drugs CPM and DXP that deserve systematic validation: (i) the bulky chloro group on C3/C7 of CPM with a strong H-bond acceptor in the β-CD–CPM inclusion complex could differ structurally and energetically from other β-CD–TCA complexes; (ii) the central seven-membered ring upon replacing C11 with hetero-atom O11 and the side chain having a C=C double bond might cause the change in inclusion structure and could differentiate the intermolecular interactions in the β-CD–*E*-DXP and the β-CD–*Z*-DXP complexes. To rationally verify the two assumptions, we carried out atomistic structural study by single-crystal X-ray diffraction and DFT calculation on the β-CD–CPM and the β-CD–DXP inclusion complexes. We also scrutinized the inclusion topologies in the six β -CD–TCA crystal structures. On top of that, a comprehensive structural comparison of the six TCAs in different lattice environments (from the uncomplexed HCl form, during delivery in the CD cavity, and to the bound state in the protein binding site) should give an in-depth understanding of the TCA structural adaptation for the pharmacological activity and the potential application of CD in drug delivery system. Thus, the study series of the β-CD–TCA complexes is fulfilled by this third paper.

## 2. Results and Discussion

**Figure 1:**
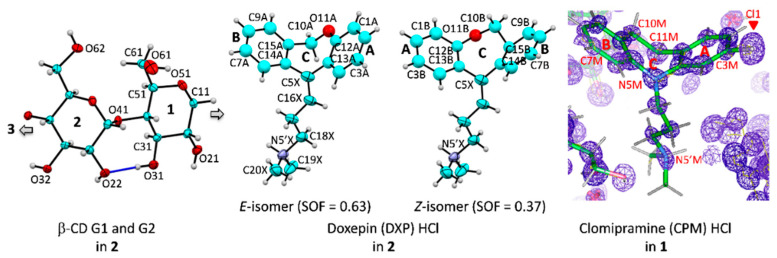
Atom numbering schemes of β-CD, DXP HCl, and CPM HCl; Oak Ridge thermal ellipsoid plot (ORTEP) plots at 30% probability level. In **1** and **2**, CPM and DXP are protonated at N5′ and counterbalanced by twofold disordered chlorides (not shown here). (**Left**) The connecting blue lines indicate the intramolecular, interglucose O3(*n*)**⋯**O2(*n* + 1) hydrogen bonds stabilizing the round β-CD conformation; see also Figure 2, Figure 3 and Figure 4. (**Middle**) For **2**, the DXP tricyclic moiety is doubly disordered with sites A and B having site occupancy factors (SOFs) of 0.63 and 0.37 for the *E* and the *Z* isomers, respectively. (**Right**) For **1**, the experimental *F*_o_ electron density map (contoured at 1.8σ) clearly shows that the chloro group is located merely on C3 of the CPM A-ring (not on C7 of the B-ring). The map was created using WinCoot [27].

Here, β-CD inclusion complexes with CPM (**1**) and DXP (**2**) are compared in detail with the other four reported complexes, including DPM (i), IPM (ii) [15], NRT (iii), and AMT (iv) [14]. As in our previous works, the nomenclature of carbohydrates is used conventionally, i.e., atoms C62–O62 indicate the methylene C6–H_2_ connected with the hydroxyl O6–H groups of glucose unit 2 (G2) in the β-CD–DXP HCl complex (**2**), Figure 1. Atom numberings of CPM and DXP are based on the corresponding IUPAC names, 3-chloro-10,11-dihydro-*N*,*N*-dimethyl-5*H*-dibenz[b,f]azepine-5 -propanamine and 11-(3-dimethyl aminopropylidene)-6,11-dihydrodibenzo[b,e]oxepine, and are further arbitrarily labeled with letters M and X, respectively (Figure 1).

For a comprehensive structural comparison of CPM HCl (**1**) and DXP HCl (**2**) with each other and with other related structures in different lattice environments, the relevant atomic coordinates were retrieved from the Cambridge Crystallographic Data Center (CCDC; www.ccdc.cam.ac.uk) [24] and the RCSB Protein Data Bank (RCSB PDB; www.rcsb.org) [25]. Surprisingly, no crystal structure of uncomplexed DXP has been reported thus far, which is probably attributed to the insufficient stability of DXP for crystal formation. Crystal structure of the human histamine H1 receptor in complex with DXP is reported at 3.1 Å resolution [26]. Here, we report the first high resolution (0.7 Å) X-ray structure of DXP in complex with β-CD, indicating the improved drug stability through CD encapsulation.

### 2.1. CDs Remain Similarly Round upon TCA Inclusion Due to the Intermolecular C–H⋯π Interactions

In the solid state, the inclusion complexes of β-CD with CPM, DXP (this work), NRT, AMT [14] and DPM, and IPM [15] belong to the same crystal symmetry (i.e., all six complexes are in the orthorhombic crystal system, space group *P*2_1_2_1_2_1_) and have similar unit cell dimensions. The asymmetric units of **1** and **2** comprise β-CD·CPM·HCl·9.6H_2_O and β-CD·DXP·HCl·0.7EtOH· 11.3H_2_O, respectively. Note that, in both complexes, CPM and DXP are protonated and are not directly coordinated by the doubly disordered chloride ion. This is recurrently observed for the crystals of TCA HCl in complex with β-CD [14,15]. However, the contrary is observed for the crystals of uncomplexed TCA HCl [24,28].

**Figure 2:**
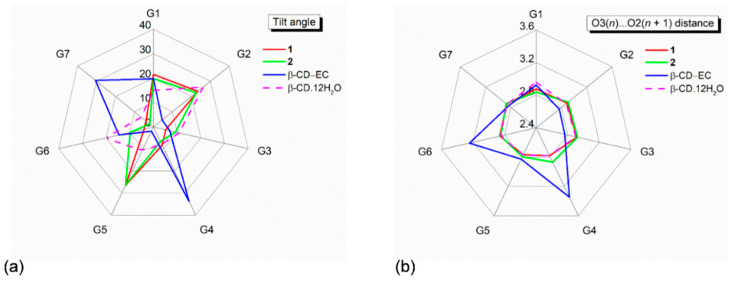
Radar plots of (**a**) tilt angles and (**b**) O3(*n*)**⋯**O2(*n* + 1) distances of the β-CD seven glucose units (G1–G7) affected by inclusion of the aromatic moieties of the CPM B-ring (**1**) and the DXP A/B-ring (**2**). For comparison, data of the inclusion complex β-CD–(−)-epicatechin(EC) [33] and the uncomplexed β-CD·12H_2_O [29] are also incorporated. Angles and distances are in ° and Å.

**Figure 3:**
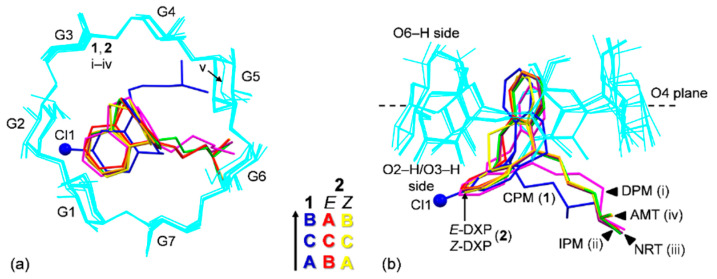
Structure overlays of six β-CDs (cyan wireframes) in complex with various TCAs (sticks), CPM (**1**; blue), DXP (**2**; *E* isomer (red), *Z* isomer (yellow)), DPM (i; magenta), IPM (ii; green), NRT (**iii**; violet), AMT (**iv**; orange) [14,15], and in the uncomplexed β-CD·12H_2_O (v) [29], viewed from (**a**) the top and (**b**) the side. Cl group on C3 (A-ring) of CPM (**1**) is emphasized with ball model. Note that the different inclusion structures from the distinct alignments of the A-C-B-rings of drugs CPM and DXP in the β-CD cavity are highlighted in the middle; see also Figure 4.

Because all the six complexes have the same crystal space group with a common crystal packing feature (a head-to-tail column) and comparable unit cell constants, β-CDs in **1** and **2** share an annular conformation with other four β-CDs in the previously reported β-CD–TCA complexes [14,15]. To quantitatively describe the structure similarity, the root mean square deviation (rmsd) of superposition of each structure pair is evaluated. All six β-CDs are similarly round, as indicated by the small rms fits of 0.028–0.108 Å, except for β-CD in complex with CPM (**1**), which is most different from the rest with several times larger values, 0.183–0.249 Å (Figure 2 and Figure 3). The non-H atoms of the β-CD skeleton excluding O6 are considered for the calculation. Comparing with the annular β-CD dodecahydrate [29], the six β-CDs are moderately affected by the inclusion of different TCAs, giving rise to distorted round forms, as indicated by larger rms fits of 0.353–0.364 Å (Figure 2 and Figure 3). The exception is β-CD (**1**) that shows maximum deviation from a round conformation, as indicated by largest rms fit of 0.491 Å. This is a paradigm of the induced-fit process [30] primarily driven by host–guest C–H**⋯**π interactions. These weak intermolecular interactions are established from the squeezing of two to three opposed glucose units at the O6–H-end to facilitate the C5–H groups to interact with the embedded TCA aromatic moiety. However, the inclusion structures of CPM and DXP are differently observed. Whereas, in **1,** the fully occupied B-ring of CPM (without substituent chloro group) is entrapped in the β-CD cavity, in **2,** the disordered A-ring of *E*-DXP (occupancy 0.63) and the B-ring of *Z*-DXP (occupancy 0.37) are enclosed in the β-CD cavity; see details in Section 2.2.

**Figure 4:**
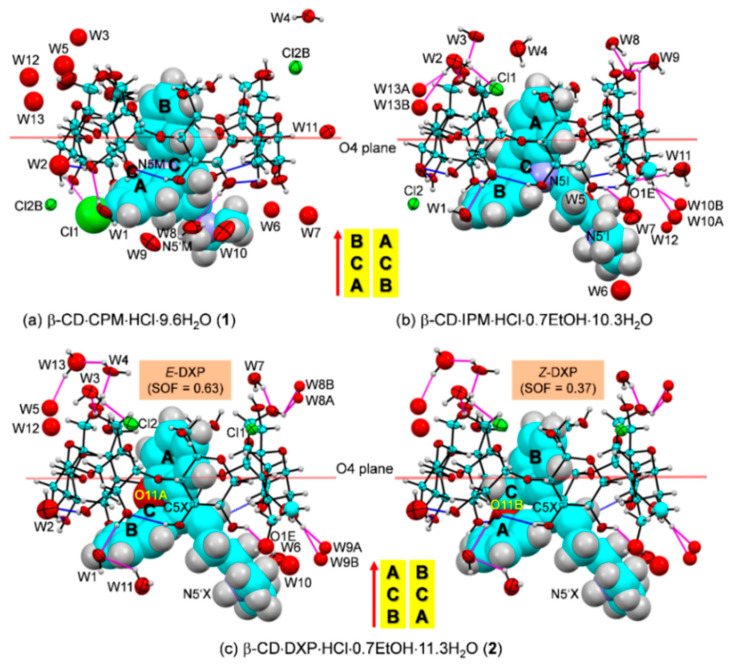
ORTEP diagrams (30% probability level) of inclusion complexes (**a**) β-CD–CPM HCl at 296 K (**1**), (**b**) β-CD–IPM HCl at 296 K [15], and (**c**) β-CD–DXP HCl at 296 K (**2**). For better visibility, the guest molecules are displayed in space-filling model. The O–H**⋯**O hydrogen bonds within β-CD and between other molecules are shown with blue and magenta connecting lines, respectively; see also Figure 5. Note that the different inclusion structures from the distinct alignments of the A-C-B-rings of drugs CPM, IPM, and DXP are emphasized in the middle; see also Figure 3.

The CD similarity stems from its structural elements. Recently, we fully described the changes in structural parameters of β-CD macrocycles upon the inclusion of TCA drugs NRT, AMT [14] and DPM, and IPM [15]. The CD structural descriptions are here briefly mentioned. The CD structural elements usually include (i) the glucose puckering parameters *Q* and θ [31], (ii) the glucose inclination angles, (iii) the deviations of glycosidic O4 atoms from their mean plane (CD molecular plane), (iv) the O4(*n*)**⋯**O4(*n* − 1), O4(*n*)**⋯**centroid distances, (v) O3(*n*)**⋯**O2(*n* + 1) distances, (vi) torsion angles φ, ψ around glycosidic O4, and (vii) torsion angles *χ*, *ω*, involving O6–H groups (Table S3). Because β-CDs in complex with CPM (**1**) and DXP (**2**) are similar to those of four reported complexes [14,15], the geometrical parameters listed in Table S3 are comparable and fall in normal ranges for the round β-CD conformation. Note that, although the belt of systematic intramolecular, interglucose O3(*n*)**⋯**O2(*n* + 1) H-bonds is broken (i.e., O32**⋯**O23, O35**⋯**O26 in **1** and O36**⋯**O27 in **2** are absent), the two β-CDs remain round (Tables S4 and S5 and Figure 2, Figure 3 and Figure 4). These O2–H, O3–H groups are engaged in H-bonding with neighboring O6–H, Cl^−^, water, ethanol molecules and C20X–H_3_ group (Tables S4 and S5). This give rises to all 14 O3(*n*)**⋯**O2(*n* + 1) distances falling in a regular range of 2.767–2.924 Å and no tilt angle exceeding 30° (Figure 2 and Table S3). By contrast, the severe distortion from a round conformation of CDs is resulted from the absence of some O3(*n*)**⋯**O2(*n* + 1) H-bonds in α-CD–*p*-nitrophenol, α-CD–*p*-hydroxybenzoic acid [32], β-CD–(−)-epicatechin(EC) [33], and the extinction of O3(*n*)**⋯**O2(*n* + 1) H-bonds in permethylated CDs, e.g., trimethyl-γ-CD hydrate [34].

The orientations of O6–H groups influenced by interactions with surroundings deserve further discussion. Note that the TCA aromatic moiety without polar substituent is entrapped in the β-CD cavity, and 9.6 and 11.3 hydration water molecules are distributed over 13 and 15 sites outside the cavity for respective complexes **1** and **2**. Consequently, 10 out of 14 O6–H groups adopt a gauche-gauche orientation and point outward the cavity to hydrogen bond with neighboring O–H groups, water sites, and chlorides (Tables S4 and S5). The corresponding torsion angles *χ*(C4–C5–C6–O6) and *ω*(O5–C5–C6–O6) are 48.5–64.7° and −55.6 to −69.5° (Table S3). O66–H (**1**), O62–H (**2**), and O66–H (**2**) groups are in a gauche-trans orientation and point toward the cavity. Moreover, the doubly disordered O63A–H and O63B–H (**1**) groups are oriented gauche-trans and gauche-gauche, respectively (Table S4).

### 2.2. The CPM Chloro Group Makes the Difference of Its Inclusion Topology Compared to Other TCA Complexes

The tricyclic cores of CPM (**1**), *E*-DXP, and *Z*-DXP (**2**) are similar to each other and to the other four complexed TCAs (NRT, AMT, DPM, and IPM), as indicated by the short spans of the butterfly angles (119.8 ± 3.1°), the annellation angles (23.8 ± 5.1°), and the A-ring-centroid–B-ring-centroid distances (4.789 ± 0.032 Å) of the embedded TCAs (Table 1). The tricyclic part is flexible to a small extent because of the quite rigid central seven-membered ring. However, the side chain portion distinguishes among various TCAs not only in the β-CD cavity but also in free HCl form and in complex with proteins. The TCA flexibility is evidenced from a number of crystal structures containing TCAs, which are conformationally compared in Section 2.4.

Thus far, there have been six crystal structures of β-CD inclusion complexes with TCAs including NRT, AMT [13,14], DPM, IPM [15] and CPM, and DXP (this work). All have a common inclusion structure of the aromatic A/B-ring, which is solely observed in the crystalline state. The inclusion of the side chain does not exist, probably due to the host–guest interactions being inappropriate and insufficient for maintaining the cohesion and the lattice stabilities. By contrast, in aqueous solution and in vacuum, the bimodal inclusion complex with the aromatic and the side chain moieties competitively bound with the β-CD cavity is frequently observed (Table S1). This is because both inclusion modes are thermodynamically stable, as theoretically demonstrated for the β-CD encapsulation of DPM and IPM [15].

**Figure 5:**
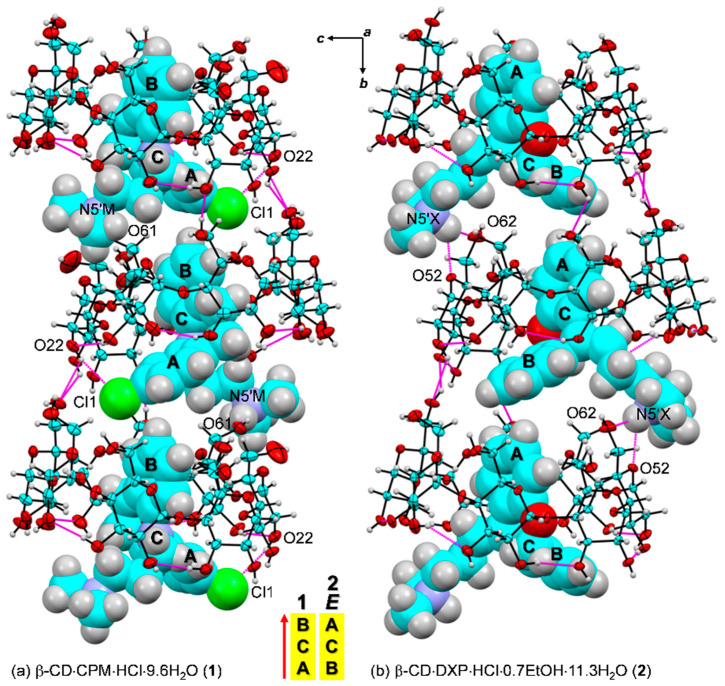
Intermolecular N/O–H**⋯**O/Cl hydrogen bonds stabilizing the inclusion complexes (**a**) β-CD–CPM HCl (**1**) and (**b**) β-CD–DXP HCl (**2**); see magenta lines. The crystal lattice is also stabilized by intermolecular edge-to-face π∙∙ π interactions between the A- and the B-rings of 2_1_-symmetry related guest molecules along the *b*-axis. The ORTEP diagrams are shown with 30% probability level. For 2, *E*-DXP (occupancy factor 0.63) is shown. Solvent molecules and chloride ions are omitted for clarity. The different inclusion structures from the varied alignments of the A-C-B-rings of CPM and DXP are emphasized in the middle.

In **1**, the B-ring (not the A-ring bearing 3-Cl group) is found to be included in the β-CD cavity, in contrast to what was previously observed for the other four crystal structures of β-CD–TCA complexes [13,14,15]. Among TCAs, from the β-CD O2–H/O3–H-side, CPM inserts the B-ring vertically and deepest, i.e., the B-ring makes an angle of 86.2° with the β-CD molecular plane, and its centroid is 1.044 Å above the O4 plane. This inclusion mode is maintained by host–guest C51/C55–H**⋯**π (B-ring) interactions (O6-end) and O22–H**⋯**Cl1, C18M–H**⋯**O34 H-bonds and C31–H**⋯**π(A-ring) interactions (O2/O3-end), Figure 4 and Figure 5, Table 2 and Table S4. Taking the lattice effect into account, the inclusion of the CPM B-ring is further stabilized by intermolecular N5′M–H**⋯**O61 H-bond and edge-to-face π**⋯**π interaction (Table 2 and Table S4). Similar host–guest interactions are observed for the β-CD encapsulation of the IPM A-ring moiety [15]. For **1**, the A-ring bearing 3-Cl group outside the β-CD cavity accepts H-bond from O22–H group, thus further stabilizing the CPM B-ring moiety in the β-CD cavity (Figure 4, Table 2 and Table S4). The chlorine atom in tricyclic CPM and halogens in SSRIs play a pivotal role in their high serotonin reuptake efficacy via polar interactions with the protein binding pockets [35,36]. The influences of Cl atom on the CPM inclusion structures and flexibilities are further theoretically explored and thoroughly discussed in respective Section 2.3 and Section 2.4.

In **2**, the inclusions of two DXP sites in the β-CD cavity are mostly identical. However, the hetero atom O11 and C5=C16 double bond distinguish the *E* from the *Z* isomers of which sites A and B have occupancy factors of 0.63 and 0.37, respectively. On comparatively stable inclusion structures, whereas *E*-DXP prefers the A-ring, *Z*-DXP favors the B-ring (Figure 4); see the DFT-derived thermodynamic data in Section 2.3. Both isomers have mostly identical inclusion structures, as the entrapped aromatic A/B-rings almost make a right angle (86.0°) against the β-CD O4 plane, and the A/B-ring centroids are 0.85, 0.71 Å above the O4 plane (Table 1 and Figure 4). The two DXP isomers are maintained in position by direct host–guest C3/C5–H**⋯**π interactions and by crystal contacts of N5′X–H**⋯**O52/O62 interactions (Figure 4 and Figure 5, Table 2 and Table S5). Insightful comparisons of the crystal structures of CPM and DXP in different circumstances are given in Section 2.4.

### 2.3. Theoretical Perspective on the β-CD Encapsulation of CPM and DXP

Supramolecular CD inclusion complexes are usually established and maintained through weak non-covalent interactions, e.g., hydrogen bonds, C/O–H**⋯**π, van der Waals, hydrophobic interactions, depending on the amphipathic CDs and the guest chemical constituents. Because the TCA drugs NRT, AMT, DPM, and IPM in active base form comprise two aromatic, non-planar fused rings and one 2°/3° alkylamine side chain, they are of hydrophobic nature. The thermodynamically stable β-CD–TCA complexes are maintained by intermolecular N/O–H**⋯**O H-bonds, C5–H**⋯**π interactions (inclusion of the aromatic A-ring), and O6–H**⋯**N5′ H-bond and O2/O3–H**⋯**π interactions (inclusion of the side chain) [14,15]. However, CPM with a substituent Cl group on the aromatic ring and DXP having the central seven-membered ring with O atom and the side chain with a C=C double bond make the inclusion scenario different, particularly in the crystalline state, as described in Section 2.2 above. To understand the structure–energy relationship of plausible inclusion modes in both complexes, full-geometry optimization by DFT calculation was performed (Section 3.3).

**Figure 6:**
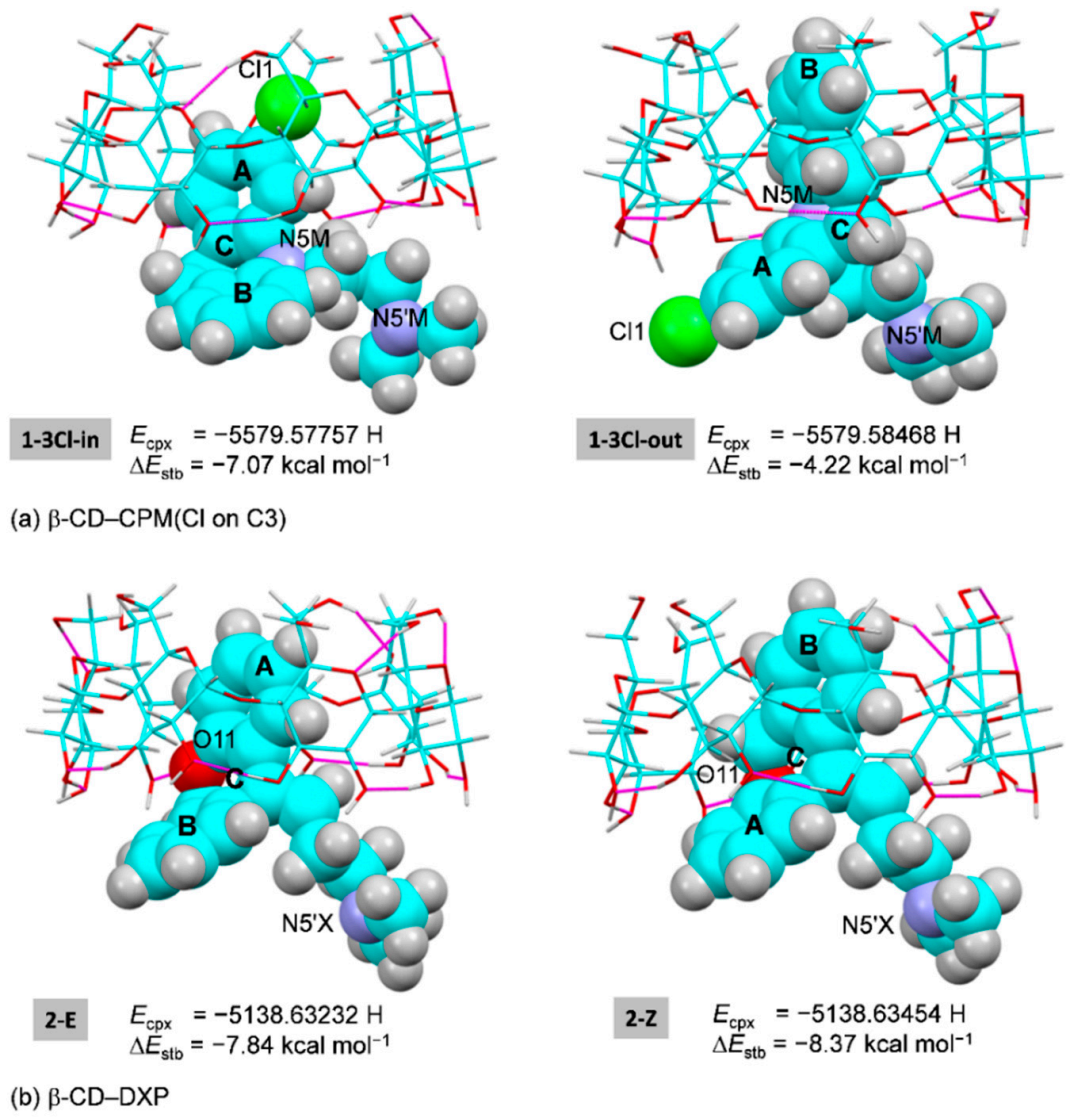
Inclusion complexes of (**a**) β-CD–CPM (Cl on C3) with Cl inside and outside the β-CD cavity, and (**b**) β-CD–*E*/*Z*-DXP, derived from DFT full-geometry optimization in the gas phase. For better comparison, the energy of complex (*E*_cpx_) and the stabilization energy (Δ*E*_stb_) are given; see also Figure 7 and Figure S1, Table 3 and Table S7 for β-CD–CPM (Cl on C7). The H-bonding interactions are indicated by the magenta lines.

In the absence of crystal contacts (i.e., solely the host–guest interactions are considered), for the β-CD–CPM complex, four inclusion modes of which the A/B-ring has a substituent Cl group on C3/C7 (i.e., **1-3Cl-in**, **1-3Cl-out**, **1-7Cl-in**, and **1-7Cl-out**) are thermodynamically plausible. This is indicated by stabilization and interaction energies (Δ*E*_stb_ and Δ*E*_int_) in the respective ranges of −4.22 to −7.07 and −6.10 to −8.37 kcal mol^−1^ Figure 6 and Figure S1, Table 3 and Table S7). The relative thermodynamic stabilities based on stabilization energy (ΔΔ*E*_stb_, kcal mol^−1^) are **1-3Cl-in** [0] > **1-7Cl-out** [1.45] > **1-7Cl-in** [1.90] > **1-3Cl-out** [2.85]. This suggests that, in the gas phase, the 3-Cl group on the A-ring embedded in the β-CD cavity (**1-3Cl-in**) is the most energetically favorable, which is in agreement with the inclusion mode observed in the four reported crystal structures of the β-CD encapsulation of NRT, AMT [14] and DPM, and IPM [15] and with the relatively high UV-Vis-derived binding constant, 9.42 × 10^3^ M^−1^ [16]. By contrast, the 3-Cl group on the A-ring outside the cavity (**1-3Cl-out**) derived from X-ray analysis is the least stable in vacuum because the 3-Cl group does not interact with β-CD (Cl1 **⋯**O22 = 4.175 Å) and hence has no contribution to the complex stability. The four inclusion modes are similarly stabilized by host–guest C3/C5–H**⋯** π and/or O2–H**⋯** π interactions (Figure 6 and Figure S1, Table 2 and Table 3, Table S7).

For the β-CD–DXP complex, the inclusions of the *E*-DXP A-ring (**2-E**) and the *Z*-DXP B-ring (**2-Z**) are comparatively stable with similar Δ*E*_stb_ and Δ*E*_int_. The small ΔΔ*E*_stb_ and ΔΔ*E*_int_ of 0.53 and 0.16 kcal mol^−1^ suggest that the β-CD–*E*-DXP is slightly less stable than the β-CD–*Z*-DXP (Table 3). Both isomeric **2-E** and **2-Z** complexes are stabilized by host–guest C3/C5–H**⋯**π interactions (Figure 6 and Figure S1, Table 2 and Table 3, Table S7). The better binding stability of *Z*-isomer compared to *E*-isomer towards the β-CD cavity helps to enhance the *Z*:*E* ratios from 0.15/0.85 (starting material) to 0.37/0.63 (refined DXP occupancy factors in the 0.70-Å resolution crystal structure). The increased quantity of the more pharmacological active *Z*-DXP [7] suggests the improved bioavailability through CD inclusion complexation. For the DXP isomers bound with the human histamine receptor, both *E* and *Z* isomers are indistinguishable at the 3.1-Å resolution and have mostly identical interactions with the binding pocket [26]. By contrast, the binding constants of 36.0 × 10^3^ and 22.7 × 10^3^ M^−1^ from capillary electrophoresis of β-CD complexes with the side chain of respective *E*- and *Z*-isomers indicate that the *E*-DXP complex is ~1.5 times more stable than the *Z*-DXP complex [20], Table S1. The improved TCA stability in the CD cavity is driven and primarily maintained by intermolecular interactions of C/O–H**⋯**π (aromatic ring embedded) and O–H**⋯**Ν, O–H**⋯**π (side chain included). The bimodal inclusion is evidenced from the single-crystal structures of six β-CD–TCA complexes and from the DFT-derived energetically favorable β-CD–DPM/IPM complexes [14,15]. This gives rise to the reduction of TCA side effects and the improvement of bioavailability via CD encapsulation [10,11,12].

**Figure 7:**
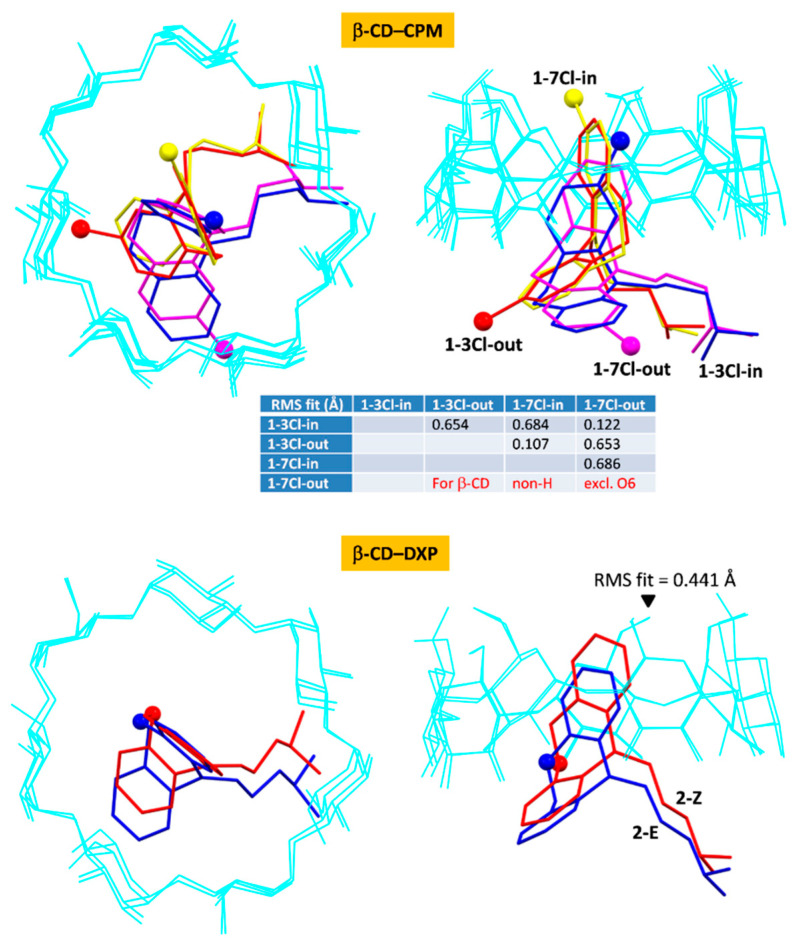
Superposition of molecular structures of β-CD–CPM (four modes) and β-CD–DXP (two modes) inclusion complexes from DFT full-geometry optimization; top view (left) and side view (right). Root mean square (RMS) fits are calculated for the host β-CDs (cyan wireframes), excluding O6, H atoms, and guest molecules; see also Figure 2 and Figure S1, Table 2 and Table 3, Table S7.

Note that β-CD adapts its conformation to an extent to form stable inclusion complexes with hydrophobic TCAs. This is indicated by rms fits of 0.107–0.686 and 0.441 Å among the four and the two modes of β-CD–CPM and β-CD–DXP complexes, respectively (Figure 7). For the DFT-derived β-CD structures of both complexes in the gas phase, the systematic intramolecular, interglucose O2(*n*)**⋯**O3(*n* + 1) H-bonds are re-established to compensate for the absence of crystal contacts and to stabilize the β-CD round conformation (Figure 3 and Figure 7 and Figure S1 and Tables S4, S5 and S7).

### 2.4. TCAs in Varied Lattice Circumstances Have High Structural Flexibility

**Figure 8:**
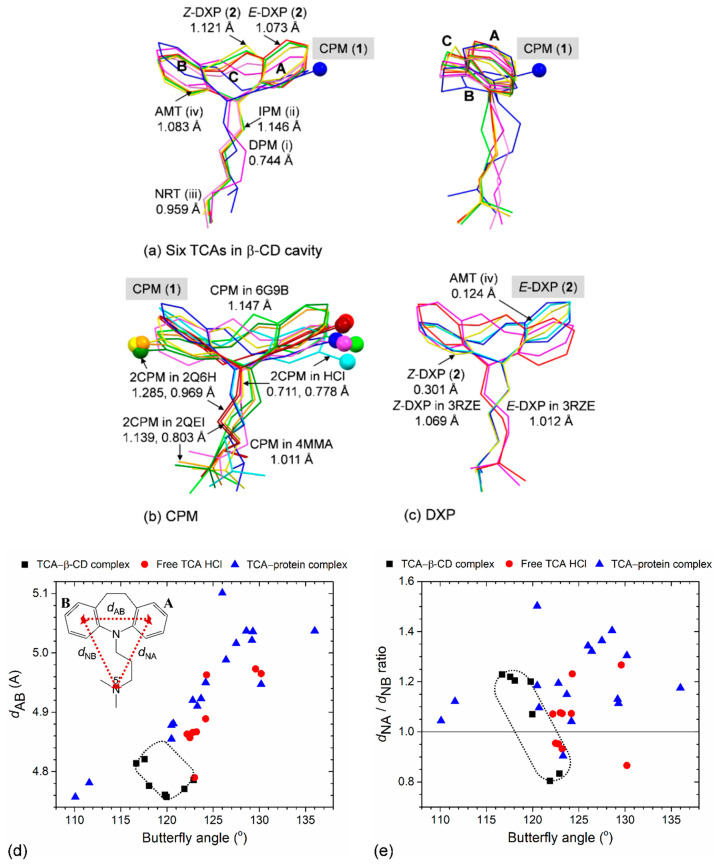
TCA structural flexibility is evidenced by comparison of six TCAs in various lattice environments (viz., TCAs in free HCl form, in β-CD cavity, and in complex with proteins (indicated by the Protein Data Bank (PDB) codes)). Structure overlays of (**a**) six TCAs including CPM (**1**), DXP (**2**), DPM (**i**), IPM (**ii**), NRT (**iii**), and AMT (**iv**) [14,15] in the β-CD cavity, viewed along the side chain (left) and along the line passing through the B–C–A rings (right), (**b**) CPM, and (**c**) DXP in different circumstances. Reference structures in (**a**–**c**) shown in blue sticks and their names marked in gray areas are used for calculating the rms fits; the corresponding value for each structure pair is indicated by nearby distance. The two CPM isomers with Cl group on C3 (A-ring) and C7 (B-ring) are emphasized with ball models. Scatter plots of (d) butterfly angle vs. *d*_AB_ distance and (**e**) butterfly angle vs. *d*_NA_/*d*_NB_ distance ratio. Data of six β-CD–TCA complexes (■) are encircled by dotted lines. An example of TCA chemical structure is also given for IPM; see inset of (**d**). For more details, see Table 1 and Table S6.

Figure 8 displays a crystallographic evidence for the TCA conformational flexibility due primarily to the alkylamine side chain moiety. This is indicated by the non-superimposable TCA structures with large rms fits (Figure 8a–c) and by the high distributions of the butterfly angle vs. the distance ratio of N5′–A-ring centroid to N5′–B-ring centroid (*d*_NA_/*d*_NB_ distance ratio), Figure 8e. In the confined β-CD cavity, both CPM (**1**) and DXP (**2**) are flexible to some extent, as previously observed in the complexes of four TCAs, including DPM, IPM [15] and NRT, and AMT [14]. The corresponding rms fits are 0.744–1.146 Å; only the TCA non-H atoms are included for the calculation, and CPM (**1**) is a reference structure (Figure 8a). This is reflected by the short spans of the butterfly angles of 116.7(3)–122.9(5)° and the A-ring-centroid–B-ring-centroid distances (*d*_AB_) of 4.757–4.821 Å and by the longer ranges of N5′–A-ring centroid and N5′–B-ring centroid distances (*d*_NA_ and *d*_NB_) of 5.479(15)–7.462(5) and 6.055(6)–7.492(8) Å, respectively (Figure 8d and Table 1).

For CPM, both positional isomers of 3-Cl and 7-Cl are found for the uncomplexed CPM HCl (code CIMPRA; [28]) and for CPM in complex with bacterial leucine transporter proteins (codes 2Q6H and 2QEI; [35]). By contrast, CPM bound with biogenic leucine transporter (code 4MMA; [36]), ebolavirus glycoprotein (code 6G9I; [37]) and encapsulated in the β-CD cavity (**1**); the isomer 3-Cl is solely observed (Table 1 and Table S6). Comparing with CPM (**1**, reference structure), the rms fits for various CPM molecules fall in the range of 0.711–1.285 Å (Figure 8b). The butterfly angles of CPM in various environments are largely fluctuated, 120.7–136.0° (Table 1 and Table S6). This is to facilitate both portions of CPM structure, including Cl atom to optimize hydrophobic and polar interactions with surrounding amino acid residues in the protein binding pocket. For DXP, both *E* and *Z* isomers embedded in the β-CD cavity (**2**) are similar to AMT [14], as indicated by the small rms fits of 0.124 and 0.249 Å (Figure 8c). By contrast, both *E*-DXP and *Z*-DXP (**2**) show greater differences from those in complex with human histamine H_1_ (code 3RZE; [26]); the large rms fits of 1.012–1.137 Å are due to the distinct structural parameters in both tricyclic core and side chain (Figure 8c and Table 1 and Table S6).

Combining the pictures from TCAs in free HCl form in complex with carrier (β-CD cavity) to TCAs in action (bound with protein binding site), the TCA flexibility is more pronounced in both portions of TCA structures (Table 1 and Table S6). This is evidenced from the large spans of the *d*_NA_/*d*_NB_ distance ratio (0.80–1.50) and the bending angle (110.1–136.0°), Figure 8e. Note that the greater N5′–A-ring centroid over N5′–B-ring centroid distances, i.e., the *d*_NA_/*d*_NB_ distance ratios greater than 1.0, indicate that the side chain is folded over the side (the B-ring), resembling the scorpion-tail orientation, as theoretically predicted two decades earlier [3]. TCA structural flexibility plays a crucial role in the formation of stable CD inclusion complexes and in the pharmacological function when in complex with proteins. The thermodynamic stabilities of plausible inclusion modes of both β-CD–CPM and β-CD–DXP complexes are theoretically evaluated in Section 2.3 above.

## 3. Materials and Methods

### 3.1. Materials

β-CD (≥95%) was obtained from Cyclolab, Budapest, Hungary (code CY-2001). CPM HCl (≥98%) and DXP HCl (≥98%; mixture of 85% *E*- and 15% *Z*-isomers) were provided by Sigma and TCI (codes C7291 and D4626), respectively. Absolute EtOH (≥99.8%) was supplied by Liquor Distillery Organization, Excise Department, Thailand. All chemicals were used as received. The ultrapure water was obtained from a Milli-Q Water System.

### 3.2. Single-Crystal Structure Determination

#### 3.2.1. Crystallization

As described in our previous works [14,15], the concentrated, homogenous solutions of the equimolar β-CD–CPM (**1**) and β-CD–DXP (**2**) inclusion complexes were prepared by dissolving β-CD 50 mg (0.044 mmol), CPM HCl 15.5 mg (0.044 mmol), and DXP HCl 13.9 mg (0.044 mmol) in 500 μL of 50% (*v*/*v*) EtOH–H_2_O at 323 K. Slow solvent evaporation in an air-conditioned room (298 K) took place for two weeks, yielding good quality single crystals suitable for X-ray analysis.

#### 3.2.2. X-ray Diffraction Experiment

X-ray diffraction data of **1** and **2** were collected at 296(2) K to respective atomic resolutions of 0.83 and 0.70 Å on a Bruker APEXII CCD area-detector diffractometer (MoKα radiation; *λ* = 0.71073 Å). Data processing was carried out with the help of the APEX2 software suite [38], i.e., the processing was begun with integration using SAINT [39], followed by scaling and multi-scan absorption correction using SADABS [38], and completed by merging with XPREP [39]. This yielded 15,560 and 25,417 independent reflections with *R*_int_ of 0.0592 and 0.0350 for the respective complexes **1** and **2**.

Several data sets of **1** were collected from crystals harvested from different crystallization batches. Various crystals of **1** gave diffraction data to ~0.8 Å resolution and the same inclusion structure, suggesting that the existence of CPM 3-chloro isomer was not pertinent to the crystal. The inclusion mode is such that the B-ring moiety is entrapped in the β-CD cavity and directed to the O6-side, and the 3-chloro group on the A-ring portion is outside the cavity, nearby the O2/O3-side. Similarly, for **2**, a few crystals were checked, and the disordered *E*- and *Z*-DXP embedded in the β-CD cavity was confirmed (see Section 2.2).

#### 3.2.3. Structure Solution and Refinement

The structures of **1** and **2** were solved by intrinsic phasing method with SHELXTL XT [38], providing all non-H atoms of β-CD, CPM, and DXP. The chloro group was merely found on C3 of the CPM A-ring, not on C7 of the B-ring (see the electron density map in Figure 1). Note that two CPM positional 3-chloro and 7-chloro isomers existed in the solid state, e.g., both isomers of uncomplexed CPM HCl crystallized in the centrosymmetric monoclinic, *P*2_1_/*c* [28], and one CPM isomer co-crystallized with proteins in non-centrosymmetric space groups; see Section 2.4 for a detailed structural comparison.

The remaining water O atoms, ethanol C, O atoms, and chloride ion were located by difference Fourier electron density maps. Anisotropic refinement by full-matrix least-squares on *F*^2^ was carried out for most of the non-H atoms using SHELXTL XLMP [38]. Exceptions are some non-H atoms of CPM (**1**), the tricyclic core of disordered DXP isomers (**2**), and some water and ethanol molecules that were refined isotropically. All H-atom positions (excluding those of OH groups) were calculated geometrically and treated with a riding model: C–H = 0.93 Å, *U*_iso_ = 1.2*U*_eq_(C)(aromatic); C–H = 0.98 Å, *U*_iso_ = 1.2*U*_eq_(C)(methine); C–H = 0.97 Å, *U*_iso_ = 1.2*U*_eq_(C)(methylene), C–H = 0.96 Å, *U*_iso_ = 1.5*U*_eq_(C)(methyl); and N–H = 0.98 Å, *U*_iso_ = 1.2*U*_eq_(3° ammonium). H atoms of hydroxyl groups and many water sites were initially located by difference Fourier maps. Then, the hydroxyl H-atoms were refined using “AFIX 147” or “AFIX 83” with restraints O–H = 0.84 Å, *U*_iso_ = 1.5*U*_eq_(O). Water H-atoms were refined with DFIX restraints to idealized geometry (O–H 0.96 Å and H**⋯**H 1.52 Å) and with ‘AFIX 3′ constraint *U*_iso_ = 1.5*U*_eq_(water). To prevent short H**⋯**H distances in the refinement, BUMP antibumping restraints were applied. The refinement converged to final *R*_1_ values of 0.1032 (**1**) and 0.0825 (**2**). Note that CPM (**1**) was found to be well ordered. For DXP (**2**), whereas the side chain was fully occupied, the tricyclic core was twofold disordered with refined occupancy factors of 0.63 (site A; *E*-isomer) and 0.37 (site B; *Z*-isomer). The increased *Z*:*E* ratios from 0.15/0.85 to 0.37/0.63 indicated the better binding to the β-CD cavity of *Z*-DXP as compared to *E*-DXP, in agreement with stabilization energies deduced from DFT calculation; see Section 2.3 and Section 3.3. For more details of data collection and refinement statistics, see Table S2.

### 3.3. DFT Full-Geometry Optimization

DFT calculation has proven to provide meaningful, reliable structures and thermodynamic data of CD inclusion complexes, as successfully demonstrated in our previous works, e.g., the β-CD encapsulation of tea catechins [40], coffee polyphenols [41], and TCA drugs [14,15]. Based on the X-ray-derived inclusion structures of **1** and **2** together with two positional isomers of CPM and *cis–trans* isomers of DXP, we considered four and two inclusion modes with TCA in active base form for DFT calculation; see the chemical structures in Scheme 1. The four modes of the β-CD–CPM base complex, namely, **1-3Cl-in**, **1-3Cl-out** (X-ray structure of **1**), **1-7Cl-in**, and **1-7Cl-out**, denoted the two isomers of CPM with Cl group on C3 (A-ring) and C7 (B-ring) residing inside or outside the β-CD cavity. For the β-CD–DXP base complex, the two modes of **2-E** and **2-Z** corresponding to the complexes of two DXP isomers were considered.

The starting inclusion structure from X-ray analysis gave a global energy minimum structure (had no negative vibrational frequency) in reasonable computing time. However, the X-ray-derived hydrogen distances were underestimated. Hence, all the involved C–H, N–H, and O–H distances were corrected to neutron hydrogen distances, 1.083, 1.009, and 0.983 Å, respectively [42], before the calculation. The initial atomic coordinates of the inclusion modes **2-E** and **2-Z** with DXP base were taken from the final structure refinement of **2** (β-CD–*E*/*Z*-DXP HCl), excluding water, ethanol molecules, and HCl. For the β-CD–CPM base complex, the inclusion modes **1-3Cl-out** and **1-7Cl-in** of which the B-ring portion was entrapped in the β-CD cavity were adapted from X-ray structure of **1** (β-CD–CPM HCl), whereas the inclusion modes **1-3Cl-in** and **1-7Cl-out** of which the A-ring portion was enclosed in the cavity were modified from X-ray structure of β-CD–DPM HCl [15]. After X–H distance normalization, the corrected structures were optimized by semiempirical PM3 method and then fully re-optimized by DFT calculation using the Becke3–Lee–Yang–Parr (B3LYP) functional in the gas phase with mixed basis sets 6-31 + G(d) for H, N, O, Cl, and 4-31G for C. All calculations were carried out using program GAUSSIAN09 [43] on a DELL PowerEdge T430 server. Stabilization energy and interaction energy of the complex (Δ*E*_stb_ and Δ*E*_int_) were calculated using Equations (1) and (2).

Δ*E*_stb_ = *E*_cpx_ − (*E*_β-CD_opt_ + *E*_D_opt_) (1)

Δ*E*_int_ = *E*_cpx_ − (*E*_β-CD_sp_ + *E*_D_sp_) (2)

where *E*_cpx_, *E*_β-CD_opt,_ and *E*_D_opt_ are the molecular energies from full-geometry optimization of complex, host β-CD, and drug CPM/DXP, respectively; *E*_β-CD_sp_ and *E*_D_sp_ are the corresponding single-point energies in the complexed states. The DFT results are summarized in Figure 6 and Figure 7, Table 2 and Table 3; see more details in Figure S1 and Table S7.

## 4. Conclusions

Depression is a global crisis of human mental health. About 300 million people of all ages (4% of the world’s population) have depression [1]. Tricyclic antidepressants (TCAs) are commonly used for effective treating depression, though they have side effects. Cyclodextrin (CD) inclusion complexation has been potentially applied for reducing the side effects and improving stability and bioavailability of TCAs. A systematic study of the β-CD–TCA complexes through single-crystal X-ray diffraction and DFT calculation aimed at atomistic understanding of the inclusion complexation. This third paper reports the β-CD encapsulation of clomipramine (CPM; **1**) and doxepin (DXP; **2**), two key drugs with high clinical efficacy. It also marks the occasion of the upcoming World Mental Health Day 2020 (10 October 2020).

Because the six key TCA drugs, including CPM, DXP, DPM, IPM, NRT, and AMT, are structurally related, they co-crystallize with β-CD, giving rather stable, equimolar inclusion complexes with the same crystal symmetry of the orthorhombic system, space group *P*2_1_2_1_2_1,_ and comparable unit cell constants. In the crystals of all complexes, the host β-CDs are packed in the head-to-tail column structure and are intact by the inclusion of various TCAs. Thus, most TCAs have the same inclusion structure in the β-CD cavity [14,15], i.e., they insert the A-ring from the β-CD O2/O3 side and almost make a right angle against the β-CD O4 plane to establish intermolecular interactions of C5–H**⋯**π(A-ring) at O6 side and/or C3–H**⋯**π(B-ring) at O2/O3 side, yielding energetically favorable inclusion complexes. Because the two TCA wings (viz., aromatic A- and B-rings) are similar, a distinct inclusion topology of the B-ring does also exist, as evidenced crystallographically in **1** and **2**. Whereas, in **1,** the 3-Cl group of CPM further stabilizes the complex via O2–H**⋯**Cl interaction, in **2,** the hetero atom O11 of *E*-DXP and *Z*-DXP has no intermolecular interaction with β-CD. The thermodynamic stabilities of both complexes evaluated using DFT calculation indicate the essence of intermolecular C/O–H**⋯**π interactions in stabilizing the β-CD–TCA inclusion complexation and thus their potential use in drug delivery systems [12]. Because the second-generation antidepressants, SSRIs, have varied structural moieties and are more flexible, their inclusion complexation attracts attention and deserves further exploration in future work.

## Figures and Tables

**Table 1:**
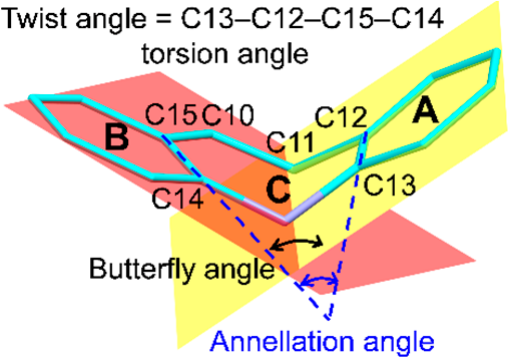
Insightful comparisons of inclusion geometries and structural parameters of six TCAs encapsulated in the β-CD cavity, CPM (**1**), *E*-, *Z*-DXP (**2**), DPM (i), IPM (ii), NRT (iii), and AMT (iv). ^a^

β-CD–TCA Complexes	CPM (1)	*E*-DXP (2) ^b^	*Z*-DXP (2) ^b^	DPM (i) ^c^	IPM (ii) ^c^	NRT (iii) ^c^	AMT (iv) ^c^
(1) Inclusion geometry							
Interplanar angle [°]							
A-ring vs. β-CD O4 plane	29.5(3)	86.0(4)	28.1(6)	83.7(2)	86.3(5)	88.4(2)	87.7(2)
B-ring vs. β-CD O4 plane	86.2(4)	24.3(3)	86.0(6)	25.8(1)	26.2(3)	25.1(1)	25.4(1)
Rings embedded in the β-CD cavity	B, C	A, C	B, C	A, C	A, C	A, C	A, C
Distance from TCA to β-CD [Å]							
A/B-ring centroid to O4 centroid (diagonal) ^d^	1.044	0.847	0.712	0.467	0.783	0.822	0.865
A/B-ring centroid to O4 plane (vertical)	1.042	0.845	0.710	0.464	0.781	0.822	0.864
C-ring centroid to O4 centroid	–1.590	–1.748	–1.908	–2.295	–1.844	–1.819	–1.791
B/A-ring centroid to O4 centroid	–3.900	–3.995	–4.134	–4.475	–4.099	–4.111	–4.081
(2) Structural parameters							
(6-7-6)-Tricyclic core							
Butterfly angle [°] ^e^	122.9(5)	118.1(6)	121.9(9)	120.0(3)	119.8(6)	116.7(3)	117.6(2)
Annellation angle [°] ^f^	28.5(6)	18.7(5)	26.4(9)	28.9(3)	26.1(6)	24.5(3)	24.5(3)
Twist angle [°] ^g^	–7.8(5)	10.3(5)	–11.5(8)	–8.1(5)	3.6(6)	4.7(4)	4.7(4)
C15–C10–C11/O11–C12 torsion angle [°]	–56.9(23)	61.5(10)	69.9(14)	–53.7(12)	43.2(32)	44.1(16)	50.4(12)
A-ring-centroid–B-ring-centroid distance *d*_AB_ [Å]	4.787	4.775	4.771	4.757	4.761	4.814	4.821
Side chain at N5/C5							
C13–N5/C5–C16–C17 torsion angle [°]	–69.4(16)	173.8(8)	4.4(15)	–64.3(7)	155.5(21)	174.4(5)	174.7(5)
N5/C5–C16–C17–C18 torsion angle [°]	–63.4(17)	136.0(9)	136.0(9)	–171.9(5)	174.6(24)	140.0(6)	138.9(5)
N5′–A-ring centroid distance *d*_NA_ [Å]	5.479(15)	7.427(8)	6.026(12)	6.564(6)	7.301(19)	7.441(6)	7.462(5)
N5′–B-ring centroid distance *d*_NB_ [Å]	6.580(14)	6.167(9)	7.492(8)	6.136(6)	6.083(20)	6.055(6)	6.118(5)

^a^ All TCAs are in HCl form. For comparison to other TCAs in free HCl form and in complex with proteins, see Table S6. ^b^ *E* and *Z* isomers of DXP have respective occupancy factors of 0.63 (site A) and 0.37 (site B). ^c^ β-CD inclusion complexes with DPM, IPM [15], and NRT, AMT [14]. ^d^ When the β-CD O6-side pointing upwards, the positive(negative) values indicate that the A/B/C-ring centroid is above (beneath) the O4 plane. ^e–g^ Interplanar angle of the A- and the B-rings; angle between C13–C12 and torsion angle C14–C15; C13–C12–C15–C14 (see inset for atom numbering).

**Table 2:** Selected host–guest interactions in the β-CD–CPM and the β-CD–DXP inclusion complexes deduced from X-ray analysis and DFT full-geometry optimization [Å, ^o^].

Interaction ^a^	D–H	H⋯A	D⋯A	∠(DHA)	Interaction ^a^	D–H	H⋯A	D⋯A	∠(DHA)
X-ray analysis					DFT calculation ^d^				
β-CD–CPM HCl (**1**) ^b^					β-CD–CPM base				
O22–H**⋯**Cl1	0.82	2.72	3.504(10)	159.5	**1-3Cl-in**				
C18M–H2**⋯**O34	0.97	2.40	3.36(2)	170.2	C31–H**⋯***Cg*1 ^e^	1.10	3.55	4.62	163.7
N5′M–H**⋯**O61 ^(i), c^	0.98	1.82	2.73(2)	152.7	O21–H**⋯***Cg*2	0.98	2.84	3.47	123.1
C31–H**⋯***Cg*1 ^d^	0.98	3.11	4.021	155.9	**1-3Cl-out**				
C51–H**⋯***Cg*2	0.98	3.75	4.542	139.5	C52–H**⋯***Cg*2	1.10	3.46	4.50	157.7
C55–H**⋯***Cg*2	0.98	3.49	4.431	162.2	C55–H**⋯***Cg*2	1.10	3.36	4.36	151.6
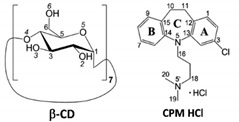					**1-7Cl-in**				
					C52–H**⋯***Cg*2	1.10	3.59	4.67	167.5
					C55–H**⋯***Cg*2	1.10	3.48	4.43	145.6
					C32–H**⋯***Cg*1	1.10	3.57	4.55	150.2
					**1-7Cl-out**				
					O21–H**⋯***Cg*2	0.98	3.11	3.69	119.9
β-CD–DXP HCl (2)					β-CD–DXP base				
N5′X–H**⋯**O52 ^(i)^	0.98	2.42	3.167(6)	132.3	**2-E**				
N5′X–H**⋯**O62 ^(i)^	0.98	2.20	3.011(9)	139.2	C31–H**⋯***Cg*2 ^f^	1.10	3.75	4.62	137.8
C31–H**⋯***Cg*2 ^e^	0.98	3.14	4.007	149.0	**2-Z**				
C31–H**⋯***Cg*3	0.98	2.95	3.837	150.7	C51–H**⋯***Cg*2	1.10	3.41	4.46	167.5
C51–H**⋯***Cg*1	0.98	3.65	4.524	149.3	C55–H**⋯***Cg*2	1.10	3.64	4.57	145.6
C55–H**⋯***Cg*1	0.98	3.32	4.244	146.9	C31–H**⋯***Cg*1	1.10	3.50	4.62	137.8
C51–H**⋯***Cg*4	0.98	3.75	4.600	157.8	C32–H**⋯***Cg*1	1.10	3.62	4.55	150.2
C55–H**⋯***Cg*4	0.98	3.46	4.400	162.7					

^a^ For full lists of intermolecular interactions, see Tables S4 and S5 (X-ray), and S6 (DFT). ^b^ For atom numbering schemes of β-CD and TCA HCl, see inset. ^c^ Symmetry-related molecule with equivalent position: (i) –*x* + 1, *y* + 0.5, –*z* + 1.5. ^d^ For stabilization and interaction energies and definitions of various inclusion modes, see Table 3. ^e^ CPM aromatic centroids: *Cg*1 = A-ring (C1M–C2M–C3M–C4M–C13M–C12M), *Cg*2 = B-ring (C6M–C7M–C8M–C9M–C15M–C14M) ^f^ DXP aromatic centroids: *Cg*1 = A-ring (C1A–C2A–C3A–C4A–C13A–C12A), *Cg*2 = B-ring (C6A–C7A–C8A–C9A–C15A–C14A) for *E* isomer *Cg*3 = A-ring (C1B–C2B–C3B–C4B–C13B–C12B), *Cg*4 = B-ring (C6B–C7B–C8B–C9B–C15B–C14B) for *Z* isomer.

**Table 3:** Stabilization and interaction energies of β-CD inclusion complexes with isomeric CPM and DXP from DFT full-geometry optimization. ^a.^

	β-CD–CPM ^b^				β-CD–DXP ^c^	
	1-3Cl-in	1-3Cl-out	1-7Cl-in	1-7Cl-out		
	A-Ring inside	A-Ring outside	B-Ring inside	B-Ring outside	2-E	2-Z
*E*_cpx_^d^	−5579.57757	−5579.58468	−5579.59119	−5579.57582	−5138.63232	−5138.63454
*E*_β-CD_opt_^e^	−4272.96719	−4272.98021	−4272.98455	−4272.96728	−4272.96876	−4272.97420
*E*_D_opt_	−1306.59911	−1306.59775	−1306.59839	−1306.59958	−865.65107	−865.64700
*E*_β-CD_sp_	−4272.96548	−4272.97744	−4272.98164	−4272.96586	−4272.96787	−4272.97340
*E*_D_sp_	−1306.59875	−1306.59752	−1306.59817	−1306.59934	−865.64185	−865.63828
Δ*E*_stb_ (Hartree) ^f^	−0.01127	−0.00673	−0.00824	−0.00896	−0.01249	−0.01334
Δ*E*_stb_ (kcal mol^–1^)	−7.07	−4.22	−5.17	−5.62	−7.84	−8.37
ΔΔ*E*_stb_ (kcal mol^–1) h^	0	2.85	1.90	1.45	0.53	0
Δ*E*_int_ (Hartree) ^g^	−0.01334	−0.00972	−0.01138	−0.01062	−0.02261	−0.02287
Δ*E*_int_ (kcal mol^–1^)	−8.37	−6.10	−7.14	−6.67	−14.19	−14.35
ΔΔ*E*_int_ (kcal mol^–1^) ^i^	0	2.27	1.23	1.70	0.16	0
Host-guest interactions	C–H**⋯**π O–H**⋯**π	C–H**⋯**π	C–H**⋯**π	O–H**⋯**π	C–H**⋯**π	C–H**⋯**π

^a^ DFT/Becke3–Lee–Yang–Parr(B3LYP) calculation using mixed basis sets 4–31G for C atoms and 6–31+G(d) for H, N, O, and Cl atoms. X-ray-derived structures are used as starting models; see also Figure 6 and Figure 7, Figure S1 and Table S7. ^b^ Four modes of the β-CD–CPM base complex, **1-3Cl-in**, **1-3Cl-out** (X-ray structure of **1**), **1-7Cl-in**, and **1-7Cl-out**, denote the two isomers of CPM with Cl group on C3 (A-ring) and C7 (B-ring) residing inside or outside the β-CD cavity. ^c^ Two modes of the β-CD–DXP base complex, **2-E** and **2-Z**, correspond to the two isomers of DXP. ^d^ Original unit of *E* is Hartree (1 H = 627.5 kcal mol^–1)^. ^e^ *E*_β-CD_opt_ in vacuum of the uncomplexed β-CD·12H_2_O [29] is −4272.96662 H. ^f,g^ Stabilization energy (Δ*E*_stb_ = *E*_cpx_ − (*E*_β-CD_opt_ + *E*_D_opt_))and interaction energy (Δ*E*_int_ = *E*_cpx_ − (*E*_β-CD_sp_ + *E*_D_sp_)), where *E*_cpx_, *E*_β__-CD_opt_ and *E*_D_opt_ are the molecular energies from full optimization of complex, host β-CD and drug CPM/DXP in the free base form, respectively; *E*_β-CD_sp_ and *E*_D_sp_ are the corresponding single-point energies in the complexed states. ^h,i^ Relative stabilization energy and relative interaction energy (ΔΔ*E*_stb_ and ΔΔ*E*_int_) calculated using the most stable inclusion mode of each complex (**1-3Cl-in** and **2-Z**) as a reference.

## Supplementary Materials

The following are available online at https://www.mdpi.com/1424-8247/13/10/278/s1. Table S1: Summary of the CD–TCA inclusion complexes characterized by various techniques, Table S2. X-ray single crystal data collection and refinement statistics of 1 and 2, Table S3: Selected geometrical parameters of two β-CD macrocycles of 1and 2, in comparison with those of β-CD–(–)-epicatechin and β-CD∙12H2O, Table S4: Hydrogen bond parameters and π⋅⋅⋅πinteractions in β-CD⋅CPM⋅HCl⋅9.6H2O(1), Table S5: Hydrogen bond parameters and π⋅⋅⋅πinteractions in β-CD⋅DXP⋅HCl⋅0.7EtOH⋅11.3H2O(2), Table S6: Structural parameters of CPM, DXP, DPM, IPM, AMT and NRT in free HCl salt form and in complex with proteins, Table S7: Hydrogen bond parameters in β-CD–CPM (4 modes) and β-CD–DXP (2 modes) inclusion complexes from DFT full-geometry optimization, Figure S1: Inclusion complexes of (a) β-CD–CPM(Cl on C3), (b) β-CD–CPM(Cl on C7) with Cl inside and outside the β-CD cavity, and (c) β-CD–E/Z-DXP, derived from DFT complete-geometry optimization in the gas phase. Crystallographic data of **1** and **2** have been deposited at the Cambridge Crystallographic Data Centre (CCDC) under respective reference numbers 2011745 and 2011746.
