# Supplementary material for: Supramolecular Complexes of β-Cyclodextrin with Clomipramine and Doxepin: Effect of the Ring Substituent and Component of Drugs on Their Inclusion Topologies and Structural Flexibilities
Source: Pharmaceuticals (Basel). 2020 Sep 29;13(10):278. doi: 10.3390/ph13100278 (PMC7600898; doi:10.3390/ph13100278)
Supplement: Supplementary file 1 [file pharmaceuticals-13-00278-s001.pdf]

# Supplementary Materials

## Supramolecular Complexes of β-Cyclodextrin with Clomipramine and Doxepin: Effect of the Ring Substituent and Component of Drugs on Their Inclusion Topologies and Structural Flexibilities

Thammarat Aree*

Department of Chemistry, Faculty of Science, Chulalongkorn University, Bangkok 10330, Thailand
* Correspondence: athammar@chula.ac.th; Tel.: +66-2-2187584; Fax +66-2-2187598

**Supplementary materials available:**

**1. Literature survey**

**Table S1.** Summary of the CD–TCA inclusion complexes characterized by various techniques.

**2. Crystallographic data**

**Table S2.** X-ray single crystal data collection and refinement statistics of **1** and **2**.

**Table S3.** Selected geometrical parameters of two β-CD macrocycles of **1** and **2**, in comparison with those of β-CD–(–)-epicatechin and β-CD·12$H_2O$.

**Table S4.** Hydrogen bond parameters and π···π interactions in β-CD·CPM·HCl·9.6$H_2O$ (**1**).

**Table S5.** Hydrogen bond parameters and π···π interactions in β-CD·DXP·HCl·0.7EtOH·11.3$H_2O$ (**2**).

**Table S6.** Structural parameters of CPM, DXP, DPM, IPM, AMT and NRT in free HCl salt form and in complex with proteins.

**3. Computational data**

**Figure S1.** Inclusion complexes of (a) β-CD−CPM(Cl on C3), (b) β-CD−CPM(Cl on C7) with Cl inside and outside the β-CD cavity, and (c) β-CD−*E*/*Z*-DXP, derived from DFT complete-geometry optimization in the gas phase.

**Table S7.** Hydrogen bond parameters in β-CD–CPM (4 modes) and β-CD–DXP (2 modes) inclusion complexes from DFT full-geometry optimization.

**References**

## 1. Literature survey

**Table S1.** Summary of the CD–TCA inclusion complexes characterized by various techniques.

| Host | Guest | Ratio | Inclusion mode [a] | | $K_a$, $M^{-1}$ (Tech.) [b] | Ref. [d] |
|---|---|---|---|---|---|---|
| | | | Aromatic | Side chain | | |
| β-CD | CPM | 1:1 | ✓ A | | $9.42\times10^3$ (U) | [1] |
| HP-β-CD | CPM | 1:1 | ✓ A | | $9.58\times10^3$ (U) | [1] |
| α-CD | DXP | 1:1 | | ✓ | 140 (FI) | [2] |
| α-CD | DXP | 2:1 | ✓ A+B | | $16.5\times10^3$ (U)<br>$19.6\times10^3$ (F) | [3] |
| α-CD | DXP | 1:1 | | ✓ | ND (Tg) | [3] |
| α-CD | DXP | 1:1 | | ✓ | $0.05\times10^3$ (I) | [4] |
| β-CD | DXP | 1:1 | | ✓ | $13.21\times10^3$ (I) | [4] |
| β-CD | DXP | 1:1 | ✓ A+B | | ND (N) | [5] |
| β-CD | DXP | 3:1 | ✓ A+B | ✓ | ND (U, F) | [6] |
| β-CD | DXP | 1:1 | ✓ | | 397 (U), 624 (F) | [6] |
| β-CD | *E*-DXP | 1:1 | | ✓ | $36.0\times10^3$ (E) | [7] |
| β-CD | *Z*-DXP | 1:1 | | ✓ | $22.7\times10^3$ (E) | [7] |
| β-CD | DXP | 1:1 | ✓ A | | ND (Tg) | [8] |
| β-CD | DXP | 2:1 | ✓ A+B | | $14.7\times10^3$ (U)<br>$16.2\times10^3$ (F) | [3] |
| β-CD | DPM | 1:1 | | ✓ | $2.04\times10^3$ (C) | [9] |
| β-CD | DPM | 2:1 | ✓ A+B | | ND (F) | [10] |
| β-CD | DPM | 1:1 | ✓ | | $8.92\times10^3$ (U) | [11] |
| β-CD | DPM | 1:1 | ✓ | ✓ | 42.2 (U), 32.0 (F) | [12] |
| β-CD | DPM | 1:1 | ✓ A | | ND (X) | [13] |
| β-CD | DPM | 1:1 | ✓ A | ✓ | ND (Tg) | [13] |
| α-CD | IPM | 1:1 | | ✓ | 130 (FI) | [2] |
| α-CD | IPM | 1:1 | | ✓ | $0.08\times10^3$ (I) | [4] |
| β-CD | IPM | 1:1 | | ✓ | $8.70\times10^3$ (I) | [4] |
| β-CD | IPM | 1:1 | | ✓ | $1.50\times10^3$ (C) | [9] |
| HP-β-CD | IPM | 1:1 | ND [c] | | $0.82\times10^3$ (C) | [14] |
| | | 1:1 | ✓ | ✓ | | |
| β-CD | IPM | 1:2 | ✓ A/B | | ND (N, L) | [15] |
| | | 2:1 | ✓ A+B | | | |
| β-CD | IPM | 2:1 | ✓ A+B | | ND (Tgl) | [15] |
| β-CD | IPM | 1:1 | ✓ A | | ND (Tg) | [8] |
| β-CD | IPM | 1:1 | | ✓ | 16.7 (U), 808 (F) | [16] |
| β-CD | IPM | 1:1 | ✓ A | | ND (X) | [13] |
| β-CD | IPM | 1:1 | ✓ A | ✓ | ND (Tg) | [13] |
| α-CD | NRT | 1:1 | | ✓ | 70 (FI) | [2] |
| α-CD | NRT | 1:1 | | ✓ | $0.09\times10^3$ (I) | [4] |
| β-CD | NRT | 1:1 | | ✓ | $16.77\times10^3$ (I) | [4] |
| β-CD | NRT | 1:1 | ✓ | ✓ | 235 (U), 211 (F) | [17] |
| β-CD | NRT | 1:1 | ✓ A | | ND (X) | [18] |
| β-CD | NRT | 1:1 | | ✓ | ND (Tgl) | [18] |
| α-CD | AMT | 1:1 | | ✓ | 113 (FI) | [2] |
| α-CD | AMT | 1:1 | | ✓ | $0.06\times10^3$ (I) | [4] |
| β-CD | AMT | 1:1 | | ✓ | $23.90\times10^3$ (I) | [4] |
| β-CD | AMT | 1:1 | | ✓ | $3.19\times10^3$ (C) | [9] |
| HP-β-CD | AMT | 1:1 | ND | | $1.03\times10^3$ (C) | [14] |
| β-CD | AMT | 1:1 | ✓ A | | ND (X) | [19] |
| β-CD | AMT | 1:1 | ✓ A | | ND (X) | [18] |
| β-CD | NRT | 1:1 | | ✓ | ND (Tgl) | [18] |

[a] TCA moiety included in CD cavity: aromatic rings A, B or side chain.
[b] Binding constant ($K_a$) at 298 K derived from different techniques, mostly in solution: flow injection (FI); fluorescence (F); UV-vis (U); ion-selective electrode (I); capillary electrophoresis (E); light scattering (L); nuclear magnetic resonance (N); theoretical calculation in gas phase or solution (Tgl); single-crystal X-ray analysis (X); conductivity (C)
[c] ND – not determined
[d] Full reference list is given on pages 15–16.

## 2. Crystallographic data

**Table S2.** X-ray single crystal data collection and refinement statistics of **1** and **2**.

| | **1**<br>β-CD–Clomipramine HCl | **2**<br>β-CD–Doxepin HCl |
|---|---|---|
| Abbreviated formula | β-CD·CPM·HCl·9.6$H_2O$ | β-CD·DXP·HCl·0.7EtOH·11.3$H_2O$ |
| Chemical formula | $(C_6H_{10}O_5)_7 \cdot C_{19}H_{23}ClN_2 \cdot HCl \cdot 9.6H_2O$ | $(C_6H_{10}O_5)_7 \cdot C_{19}H_{21}NO \cdot HCl$<br>$\cdot 0.7(C_2H_6O) \cdot 11.3H_2O$ |
| Formula weight | 1645.32 | 1679.38 |
| Crystal habit, color | Thin plate, colorless | Hexagonal, colorless |
| Crystal size [mm] | 0.04 × 0.26 × 0.38 | 0.30 × 0.42 × 0.42 |
| Crystal system, space gr. | Orthorhombic, $P2_12_12_1$ (No. 19) | Orthorhombic, $P2_12_12_1$ (No. 19) |
| *a*, *b*, *c* [Å] | 15.2643(6), 18.7352(7), 29.5743(10) | 15.1521(4), 18.4814(4), 29.6212(9) |
| $\alpha$, $\beta$, $\gamma$ [°] | 90, 90, 90 | 90, 90, 90 |
| $V$ [Å$^3$] | 8457.7(5) | 8294.9(4) |
| $Z$ | 4 | 4 |
| $D_c$ [g cm$^{-3}$] | 1.292 | 1.328 |
| $\mu$ [mm$^{-1}$] | 0.171 | 0.147 |
| $F$(000) | 3481 | 3576 |
| Diffractometer | APEXII Kappa CCD (Bruker) | APEXII Kappa CCD (Bruker) |
| Wavelength [Å] | MoK$_\alpha$, 0.71073 | MoK$_\alpha$, 0.71073 |
| T [K] | 296 | 296 |
| Data collection | $\omega$–$\phi$ scan, 0.4° step, 8 s expose | $\omega$–$\phi$ scan, 0.4° step, 8 s expose |
| Frames collected | 658 | 659 |
| $\theta$ range [°] | 1.38–25.42 | 1.74–30.61 |
| Resolution [Å] | 0.83 | 0.70 |
| Completeness [%], $R_{int}$ | 99.7, 0.0592 | 99.8, 0.0350 |
| Reflns<br>collected / unique / observed | 43530 / 15560 / 7967 | 55043 / 25417 / 16493 |
| Data / restraints / parameters | 15560, 59, 956 | 16493, 44, 939 |
| $R_1$ [a], $wR_2$ $[I > 2\sigma(I)]$ [b] | 0.1032, 0.2676 | 0.0825, 0.2235 |
| $R_1$, $wR_2$ [all data], GoF | 0.1772, 0.3218, 1.005 | 0.1219, 0.2554, 1.026 |
| $\Delta\rho_{min}$, $\Delta\rho_{max}$ [e Å$^{-3}$] | –0.60, 0.82 | –0.50, 0.93 |
| CCDC number | 2011745 | 2011746 |

[a,b] $R = \sum||F_o| - |F_c||/\sum|F_o|$; $wR = \sum\{w(F_o^2 - F_c^2)^2/\sum w(F_o^2)^2\}^{1/2}$.

**Table S3.** Selected geometrical parameters of two β-CD macrocycles of **1** and **2**, in comparison with those of β-CD–(–)-epicatechin and β-CD·12$H_2O$.

| Residue | Puckering $Q$ [Å] [a], $\theta$ [°] [b] | | | | Tilt angle [°] [c] | | | | O4 deviation [Å] [d] | | | | O4($n$)···O4($n$ – 1), O4($n$)···centroid [Å] | | | |
|---|---|---|---|---|---|---|---|---|---|---|---|---|---|---|---|---|
| $n$ | **1** | **2** | **β-CD–EC** [e] | **β-CD·12W** [f] | **1** | **2** | **β-CD–EC** | **β-CD·12W** | **1** | **2** | **β-CD–EC** | **β-CD·12W** | **1** | **2** | **β-CD–EC** | **β-CD·12W** |
| 1 | 0.571(11) | 0.593(4) | 0.561(2) | 0.570 | | | | | | | | | 4.573(8) | 4.528(4) | 4.403(2) | 4.489 |
| | 9.4(11) | 8.3(5) | 6.7(2) | 7.6 | 21.5(4) | 19.8(1) | 19.7(1) | 15.0 | –0.218(5) | –0.222(2) | –0.180(1) | 0.192 | 4.713(5) | 4.781(3) | 5.171 | 4.981 |
| 2 | 0.566(10) | 0.589(4) | 0.545(2) | 0.583 | | | | | | | | | 4.419(9) | 4.410(4) | 4.263(2) | 4.392 |
| | 2.9(10) | 1.8(5) | 5.6(2) | 3.0 | 23.4(2) | 22.5(1) | 4.6(1) | 26.2 | 0.069(5) | 0.013(2) | –0.084(1) | 0.091 | 5.255(5) | 5.272(3) | 5.397 | 5.153 |
| 3 | 0.540(11) | 0.566(4) | 0.574(2) | 0.559 | | | | | | | | | 4.166(9) | 4.199(4) | 4.278(2) | 4.286 |
| | 7.7(12) | 5.8(4) | 3.7(2) | 3.9 | 5.2(2) | 9.3(1) | 6.9(1) | 10.8 | 0.270(6) | 0.254(3) | 0.325(1) | –0.195 | 5.231(6) | 5.216(3) | 4.581 | 5.122 |
| 4 | 0.556(10) | 0.579(5) | 0.557(2) | 0.596 | | | | | | | | | 4.418(8) | 4.435(4) | 4.498(2) | 4.443 |
| | 5.7(10) | 3.3(5) | 4.9(3) | 1.4 | 8.3(2) | 6.6(1) | 33.7(1) | 7.9 | –0.285(6) | –0.176(3) | –0.199(1) | –0.053 | 4.664(6) | 4.640(3) | 4.827 | 4.856 |
| 5 | 0.557(11) | 0.571(5) | 0.578(2) | 0.579 | | | | | | | | | 4.463(9) | 4.472(4) | 4.322(2) | 4.452 |
| | 5.3(10) | 4.0(5) | 3.2(2) | 2.0 | 26.5(4) | 26.4(2) | 1.9(1) | 10.7 | –0.053(5) | –0.127(3) | –0.073(1) | 0.276 | 4.892(5) | 5.006(3) | 5.435 | 5.054 |
| 6 | 0.552(11) | 0.552(4) | 0.565(2) | 0.571 | | | | | | | | | 4.283(8) | 4.244(4) | 4.209(2) | 4.247 |
| | 5.7(11) | 7.1(4) | 4.9(2) | 3.9 | 3.9(2) | 9.8(1) | 14.6(1) | 20.3 | 0.236(6) | 0.184(2) | 0.087(1) | –0.115 | 5.416(6) | 5.390(3) | 5.075 | 5.184 |
| 7 | 0.545(10) | 0.560(5) | 0.582(2) | 0.567 | | | | | | | | | 4.197(8) | 4.308(4) | 4.626(2) | 4.338 |
| | 5.7(11) | 2.2(5) | 8.8(2) | 3.7 | 3.9(2) | 1.9(1) | 30.6(1) | 6.4 | –0.020(5) | 0.074(2) | 0.124(1) | –0.196 | 4.896(6) | 4.862(3) | 4.632 | 4.913 |
| | | | | | | | | | | | | | *0.407* [g] | *0.329* | *0.417* | *0.242* |
| | | | | | | | | | | | | | *0.752* [g] | *0.750* | *0.854* | *0.328* |
| | | | | | | | | | | | | | *0.874* [g] | *0.873* | *0.876* | *0.870* |

[a,b] A perfect cyclohexane chair (for $R$(C–C) = 1.54 Å) has puckering amplitude $Q$ = 0.63 Å and angle describing the polar position $\theta$ = 0° [20].

[c] Interplanar angle of the plane through C1($n$), C4($n$), O4($n$) and O4($n$ – 1) against the O4 plane.

[d] Deviation of glycosidic O4 atoms from the least-squares plane through the seven O4 atoms.

[e] β-CD–(–)-epicatechin(EC) [21].

[f] β-CD·12$H_2O$ [22].

[g] Ranges of the O4($n$)···O4($n$ – 1), O4($n$)···centroid distances and the average of their ratios are in *italics*; for an ideal heptagon, the ratio is 0.868.

[h] Endocyclic torsion angles $\phi$ and $\psi$ at glycosidic O4, defined as O5($n$ + 1)–C1($n$ + 1)–O4($n$)–C4($n$) and C1($n$ + 1)–O4($n$)–C4($n$)–C5($n$), respectively.

[i] Averages of $\phi$ and $\psi$ are in *italics*; for the β-CD roundness, the sum of averages should be zero [23].

[j] Exocyclic torsion angles $\chi$ and $\omega$ are defined as C4–C5–C6–O6 and O5–C5–C6–O6, respectively.

[k,m] Doubly disordered O63–H group with occupancy factors 0.44 and 0.56 for respective sites A and B.

**Table S3.** Continued.

| Residue | O3(*n*)···O2(*n* + 1) distance [Å] | | | | Torsion angles $\phi$ [h], $\psi$ [h] [°] | | | | Torsion angles $\chi$ [j], $\omega$ [j] [°] | | | |
|---|---|---|---|---|---|---|---|---|---|---|---|---|
| *n* | **1** | **2** | **β-CD–EC** | **β-CD·12W** | **1** | **2** | **β-CD–EC** | **β-CD·12W** | **1** | **2** | **β-CD–EC** | **β-CD·12W** |
| 1 | 2.877(11) | 2.838(5) | 2.924(2) | 2.957 | 116.6(9) | 116.0(4) | 100.7(2) | 119.3 | 54.4(13) | 48.5(6) | 53.5(2) | –169.4 |
| | | | | | –100.2(10) | –99.8(4) | –121.5(2) | –95.9 | –64.1(11) | –69.5(6) | –66.8(2) | 70.5 |
| 2 | 2.885(10) | 2.905(5) | 2.765(2) | 2.875 | 107.0(10) | 108.7(4) | 111.6(2) | 110.5 | 56.1(17) | 175.5(5) | 58.7(3) | –173.9 |
| | | | | | –111.3(10) | –115.1(4) | –118.1(2) | –106.6 | –66.1(16) | 57.1(6) | –62.7(2) | 71.0 |
| 3 | 2.917(11) | 2.924(6) | 2.762(2) | 2.902 | 105.3(8) | 103.9(4) | 129.1(2) | 102.5 | –172.3(38) [k] 61.6(43) [m] | 58.2(5) | 48.9(2) | 58.7 |
| | | | | | –120.3(8) | –118.3(4) | –97.4(2) | –121.1 | 66.4(51) [k] –73.9(36) [m] | –63.9(5) | –72.5(2) | –60.8 |
| 4 | 2.782(12) | 2.870(7) | 3.346(3) | 2.783 | 105.7(9) | 104.3(4) | 90.1(2) | 107.7 | 56.3(11) | 56.2(5) | 55.7(3) | 57.0 |
| | | | | | –108.9(10) | –113.4(4) | –131.9(2) | –109.4 | –63.6(10) | –63.5(5) | –64.5(2) | –61.0 |
| 5 | 2.767(12) | 2.794(5) | 2.828(3) | 2.770 | 122.9(9) | 120.9(4) | 117.1(2) | 110.7 | 63.3(11) | 64.7(6) | 60.7(2) | 50.7 |
| | | | | | –104.1(10) | –101.0(5) | –105.9(2) | –114.1 | –57.6(11) | –55.6(6) | –60.9(2) | –71.0 |
| 6 | 2.862(13) | 2.850(6) | 3.246(3) | 2.855 | 105.8(9) | 104.9(4) | 105.9(2) | 120.0 | –176.0(8) | –173.9(4) | –167.8(2) | –175.4 |
| | | | | | –129.6(8) | –123.1(4) | –96.5(2) | –109.8 | 63.8(10) | 65.2(6) | 69.8(2) | 64.7 |
| 7 | 2.867(12) | 2.874(5) | 2.833(3) | 2.862 | 114.3(9) | 108.4(4) | 102.7(2) | 103.0 | 53.0(11) | 54.7(5) | 54.2(3) | 52.1 |
| | | | | | –111.8(9) | –119.0(4) | –118.4(2) | –125.7 | –69.3(10) | –66.3(5) | –64.7(3) | –62.9 |
| | | | | | *111.1* [i] | *109.6* | *108.2* | *110.5* | | | | |
| | | | | | *–112.3* [i] | *–112.8* | *–112.8* | *–111.8* | | | | |
| | | | | | *–1.2* [i] | *–3.2* | *–4.6* | *–1.3* | | | | |

[a,b] A perfect cyclohexane chair (for $R$(C–C) = 1.54 Å) has puckering amplitude $Q$ = 0.63 Å and angle describing the polar position $\theta$ = 0° [20].
[c] Interplanar angle of the plane through C1(*n*), C4(*n*), O4(*n*) and O4(*n* – 1) against the O4 plane.
[d] Deviation of glycosidic O4 atoms from the least-squares plane through the seven O4 atoms.
[e] β-CD–(–)-epicatechin(EC) [21].
[f] β-CD·12$H_2O$ [22].
[g] Ranges of the O4(*n*)···O4(*n* – 1), O4(*n*)···centroid distances and the average of their ratios are in *italics*; for an ideal heptagon, the ratio is 0.868.
[h] Endocyclic torsion angles $\phi$ and $\psi$ at glycosidic O4, defined as O5(*n* + 1)–C1(*n* + 1)–O4(*n*)–C4(*n*) and C1(*n* + 1)–O4(*n*)–C4(*n*)–C5(*n*), respectively.
[i] Averages of $\phi$ and $\psi$ are in *italics*; for the β-CD roundness, the sum of averages should be zero [23].
[j] Exocyclic torsion angles $\chi$ and $\omega$ are defined as C4–C5–C6–O6 and O5–C5–C6–O6, respectively.
[k,m] Doubly disordered O63–H group with occupancy factors 0.44 and 0.56 for respective sites A and B.

**Table S4.** (a) Hydrogen bond parameters in β-CD·CPM·HCl·9.6$H_2O$ (**1**) [Å, °].

| D–H···A | D–H | H···A | D···A | ∠(DHA) | D–H···A | D–H | H···A | D···A | ∠(DHA) |
|---|---|---|---|---|---|---|---|---|---|
| β-*CD*–β-*CD* | | | | | | | | | |
| O31–H···O22 | 0.82 | 2.10 | 2.877(11) | 158.5 | O25–H···O6W | 0.82 | 2.14 | 2.84(2) | 143.4 |
| O22–H···O66 [ii,b] | 0.82 | 2.53 | 3.043(11) | 122.0 | O35–H···O6W | 0.82 | 2.52 | 3.31(2) | 164.4 |
| O32–H···O66 [ii] | 0.82 | 2.22 | 2.860(10) | 134.7 | O26–H···O7W | 0.82 | 2.28 | 3.08(4) | 164.1 |
| O33–H···O24 | 0.82 | 2.12 | 2.917(11) | 163.6 | O66–H···Cl2B | 0.82 | 1.93 | 2.736(12) | 165.5 |
| O34–H···O25 | 0.82 | 2.01 | 2.782(12) | 157.5 | O27–H···O8W | 0.82 | 2.43 | 3.24(2) | 166.7 |
| O36–H···O27 | 0.82 | 2.26 | 2.862(13) | 130.2 | O4W–H2···O27 [vi] | 0.96 | 2.30 | 3.147(13) | 146.7 |
| O37–H···O21 | 0.82 | 2.21 | 2.867(12) | 137.4 | β-*CD*–*CPM* | | | | |
| O67–H···O33 [v] | 0.82 | 1.98 | 2.753(11) | 157.5 | O22–H···Cl1 | 0.82 | 2.72 | 3.504(10) | 159.5 |
| β-*CD*–$H_2O$/*Cl* [a] | | | | | C18M–H2···O34 | 0.97 | 2.40 | 3.36(2) | 170.2 |
| O21–H···O1W | 0.82 | 2.48 | 2.91(2) | 113.7 | N5'M–H···O61 [ii] | 0.98 | 1.82 | 2.73(2) | 152.7 |
| O61–H···O3W | 0.82 | 2.36 | 3.17(2) | 172.4 | C31–H···*Cg*1 [d] | 0.98 | 3.11 | 4.021 | 155.9 |
| O4W–H1···O22 [v] | 0.96 | 2.65 | 3.139(11) | 112.0 | C51–H···*Cg*2 | 0.98 | 3.75 | 4.542 | 139.5 |
| O62–H···O6W [i] | 0.82 | 2.19 | 2.90(3) | 144.8 | C55–H···*Cg*2 | 0.98 | 3.49 | 4.431 | 162.2 |
| O23–H···Cl2A | 0.82 | 2.05 | 2.773(10) | 146.1 | $H_2O$–$H_2O$ | | | | |
| O63B–H···O7W [i,c] | 0.82 | 2.44 | 2.93(4) | 119.3 | O1W–H2···O9W | 0.96 | 2.33 | 3.12(3) | 140.0 |
| O24–H···O9W [iii] | 0.82 | 2.04 | 2.81(2) | 157.4 | O8W–H1···O10W | 0.96 | 1.88 | 2.81(3) | 162.3 |
| O64–H···Cl2A [iv] | 0.82 | 1.91 | 2.674(9) | 155.2 | O8W–H2···O9W | 0.96 | 1.99 | 2.87(3) | 152.4 |

[a] Site occupancy factors (SOFs) are as follows
9.6 water molecules are distributed over 13 sites: 1.0 (O4W, O8W, O9W, O11W); 0.8 (O2W, O3W, O6W); 0.7 (O1W); 0.6 (O7W, O10W); 0.5 (O5W, O12W); 0.3 (O13W)
One twofold disordered chloride: 0.5 (Cl2A, Cl2B)

[b] Equivalent positions: (i) $-x + 1.5, -y + 1, z + 0.5$; (ii) $-x + 1, y + 0.5, -z + 1.5$; (iii) $x + 1, y, z$; (iv) $-x + 2, y - 0.5, -z + 1.5$; (v) $-x + 1, y - 0.5, -z + 1.5$; (vi) $x + 0.5, -y + 0.5, -z + 1$.

[c] Twofold disordered O63–H group with occupancy factors 0.44 and 0.56 for respective sites A and B.

[d] *Cg*1 = A-ring (C1M–C2M–C3M–C4M–C13M–C12M), *Cg*2 = B-ring (C6M–C7M–C8M–C9M–C15M–C14M)

(b) π···π interactions in β-CD·DPM·HCl·0.8EtOH·9.4$H_2O$ (**1**) [Å, °].

| *Cg*(*I*) | *Cg*(*J*) | *Cg*–*Cg* | Alpha | *CgI*_Perp | *CgJ*_Perp | Type |
|---|---|---|---|---|---|---|
| *Cg*1 | *Cg*2($-x + 1, y + 0.5, -z + 1.5$) | 5.877(5) | 118.7(5) | 2.209(4) | 3.654(4) | Edge-to-face |

Note:
- *Cg*(*I*) = Plane number *I*
  *Cg*1 = A-ring (C1M–C2M–C3M–C4M–C13M–C12M), *Cg*2 = B-ring (C6M–C7M–C8M–C9M–C15M–C14M)
- Alpha = Interplanar angle between planes *I* and *J* [°]
- *Cg*–*Cg* = Distance between ring centroids [Å]
- *CgI*_Perp = Perpendicular distance of *Cg*(*I*) on ring *J* [Å]
- *CgJ*_Perp = Perpendicular distance of *Cg*(*J*) on ring *I* [Å]

**Table S5.** (a) Hydrogen bond parameters in β-CD·DXP·HCl·0.7EtOH·11.3$H_2O$ (**2**) [Å, °].

| D–H···A | D–H | H···A | D···A | ∠(DHA) | D–H···A | D–H | H···A | D···A | ∠(DHA) |
|---|---|---|---|---|---|---|---|---|---|
| β-*CD*–β-*CD* | | | | | | | | | |
| O31–H···O22 | 0.82 | 2.06 | 2.838(5) | 158.1 | O65–H···O8WB | 0.82 | 2.55 | 3.12(2) | 126.7 |
| O22–H···O66 [i b] | 0.82 | 2.47 | 3.066(5) | 130.8 | O26–H···O9WA | 0.82 | 2.00 | 2.808(11) | 167.5 |
| O32–H···O23 | 0.82 | 2.10 | 2.905(5) | 169.1 | O26–H···O9WB | 0.82 | 2.00 | 2.80(2) | 164.3 |
| O33–H···O67 [i] | 0.82 | 2.28 | 2.828(5) | 124.2 | O27–H···O1E | 0.82 | 2.40 | 3.041(14) | 136.0 |
| O24–H···O33 | 0.82 | 2.14 | 2.924(6) | 160.4 | β-*CD*–*DXP* | | | | |
| O25–H···O34 | 0.82 | 2.29 | 2.870(7) | 128.2 | N5'X–H···O52 [i] | 0.98 | 2.42 | 3.167(6) | 132.3 |
| O35–H···O26 | 0.82 | 1.98 | 2.793(5) | 170.7 | N5'X–H···O62 [i] | 0.98 | 2.20 | 3.011(9) | 139.2 |
| O66–H···O32 [iv] | 0.82 | 2.07 | 2.782(5) | 144.3 | C31–H···*Cg*2 [c] | 0.98 | 3.14 | 4.007 | 149.0 |
| O37–H···O21 | 0.82 | 2.22 | 2.874(5) | 137.3 | C31–H···*Cg*3 | 0.98 | 2.95 | 3.837 | 150.7 |
| O67–H···O64 [v] | 0.82 | 2.13 | 2.920(6) | 161.1 | C51–H···*Cg*1 | 0.98 | 3.65 | 4.524 | 149.3 |
| β-*CD*–*H₂O/EtOH/Cl* [a] | | | | | C55–H···*Cg*1 | 0.98 | 3.32 | 4.244 | 146.9 |
| O21–H···O1W | 0.82 | 2.03 | 2.785(7) | 152.0 | C51–H···*Cg*4 | 0.98 | 3.75 | 4.600 | 157.8 |
| O61–H···O3W | 0.82 | 2.21 | 2.955(7) | 150.8 | C55–H···*Cg*4 | 0.98 | 3.46 | 4.400 | 162.7 |
| O62–H···O4W | 0.82 | 1.92 | 2.722(7) | 167.5 | *$H_2O$–$H_2O$/EtOH/Cl* | | | | |
| O23–H···Cl1 [i] | 0.82 | 1.97 | 2.779(5) | 168.3 | O3W–H1···O12W | 0.96 | 1.88 | 2.81(2) | 161.7 |
| O63–H···Cl2 [ii] | 0.82 | 2.05 | 2.816(6) | 154.1 | O3W–H2···Cl2 | 0.96 | 2.32 | 3.234(9) | 158.9 |
| O11W–H2···O24 [v] | 0.96 | 2.03 | 2.941(10) | 158.5 | O4W–H1···O11W [iv] | 0.96 | 1.90 | 2.786(10) | 152.5 |
| O34–H···O13W [iii] | 0.82 | 2.38 | 2.97(2) | 129.4 | O4W–H2···O13W | 0.96 | 1.95 | 2.78(2) | 143.7 |
| O64–H···Cl1 [ii] | 0.82 | 2.00 | 2.739(5) | 149.2 | O6W–H2···O12W [i] | 0.96 | 2.20 | 2.94(2) | 132.5 |
| O1E–H···O25 [v] | 0.82 | 2.41 | 2.939(13) | 123.5 | O7W–H1···Cl1 [ii] | 0.96 | 2.06 | 2.906(8) | 146.1 |
| O6W–H1···O35 | 0.96 | 2.34 | 2.819(10) | 110.4 | O11W–H1···O1W | 0.96 | 2.14 | 2.830(11) | 127.5 |
| O7W–H2···O65 | 0.96 | 1.87 | 2.807(8) | 164.0 | O13W–H1···O1E [iv] | 0.96 | 2.31 | 3.03(2) | 131.4 |
| O65–H···O8WA | 0.82 | 2.09 | 2.740(8) | 136.4 | O13W–H2···O5W | 0.96 | 1.92 | 2.82(2) | 155.5 |

[a] Site occupancy factors (SOFs) are as follows
11.3 water molecules distributed over 15 sites: 1.0 (O1W–O4W, O6W, O7W, O11W); 0.7 (O5W, O8WA, O13W); 0.6 (O9WA, O12W); 0.4 (O9WB), 0.3 (O8WB, O10W);
EtOH: 0.7 (O1E); one twofold disordered chloride: 0.5 (Cl1, Cl2)

[b] Equivalent positions: (i) $-x + 1, y + 0.5, -z + 1.5$; (ii) $x + 1, y, z$; (iii) $-x + 2, y + 0.5, -z + 1.5$;
(iv) $-x + 1, y - 0.5, -z + 1.5$; (v) $x - 1, y, z$; $-x + 1.5, -y + 1, z - 0.5$.

[c] *Cg*1 = A-ring (C1A–C2A–C3A–C4A–C13A–C12A), *Cg*2 = B-ring (C6A–C7A–C8A–C9A–C15A–C14A) for *E*-DXP
*Cg*3 = A-ring (C1B–C2B–C3B–C4B–C13B–C12B), *Cg*4 = B-ring (C6B–C7B–C8B–C9B–C15B–C14B) for *Z*-DXP

(b) π···π interactions in β-CD·DXP·HCl·0.7EtOH·11.3$H_2O$ (**2**) [Å, °].

- *E*-DXP (DXP site A with SOF = 0.63)

| *Cg*(*I*) | *Cg*(*J*) | *Cg*–*Cg* | Alpha | *CgI*_Perp | *CgJ*_Perp | Type |
|---|---|---|---|---|---|---|
| *Cg*2 | *Cg*1(–*x* + 1, *y* + 0.5, –*z* + 1.5) | 5.461(5) | 108.5(5) | 2.136(3) | 4.074(3) | Edge-to-face |

- *Z*-DXP (DXP site B with SOF =0.37)

| *Cg*(*I*) | *Cg*(*J*) | *Cg*–*Cg* | Alpha | *CgI*_Perp | *CgJ*_Perp | Type |
|---|---|---|---|---|---|---|
| *Cg*3 | *Cg*4(–*x* + 1, *y* + 0.5, –*z* + 1.5) | 5.575(8) | 117.5(8) | 1.496(5) | 4.054(5) | Edge-to-face |

Note:
- *Cg*(*I*) = Plane number *I*
  *Cg*1 = A-ring (C1A–C2A–C3A–C4A–C13A–C12A), *Cg*2 = B-ring (C6A–C7A–C8A–C9A–C15A–C14A)
  *Cg*3 = A-ring (C1B–C2B–C3B–C4B–C13B–C12B), *Cg*4 = B-ring (C6B–C7B–C8B–C9B–C15B–C14B)
- Alpha = Interplanar angle between planes *I* and *J* [°]
- *Cg*–*Cg* = Distance between ring centroids [Å]
- *CgI*_Perp = Perpendicular distance of *Cg*(*I*) on ring *J* [Å]
- *CgJ*_Perp = Perpendicular distance of *Cg*(*J*) on ring *I* [Å]

**Table S6.** Structural parameters of CPM, DXP, DPM, IPM, AMT and NRT in free HCl salt form and in complex with proteins.

(a) Free HCl salt form

| CSD/PDB code [d] | CIMPRA [e] | | PUKGEI [f] | | | | IMIPRC [g] | | JINGIW [h] | YOVZEO [i] |
|---|---|---|---|---|---|---|---|---|---|---|
| | CPM | | DPM | | | | IPM | | NRT | AMT |
| | 7-Cl [j] | 3-Cl [j] | Mol 1 [k] | Mol 2 | Mol 3 | Mol 4 | Mol 1 | Mol 2 | | |
| 1) [6-7-6]-Tricyclic core | | | | | | | | | | |
| Butterfly angle [°] [a] | 123.2 | 123.2 | 122.5 | 122.2 | 124.2 | 122.8 | 123.0 | 130.2 | 124.3 | 129.6 |
| Annellation angle [°] [b] | 32.5 | 32.5 | 32.0 | 31.1 | 32.0 | 31.8 | 26.6 | 32.5 | 29.6 | 25.7 |
| Twist angle [°] [c] | −15.5 | 15.5 | −13.3 | 13.7 | 13.9 | −13.5 | 7.4 | 15.2 | 13.8 | 7.2 |
| C15–C10–C11–C12 torsion angle [°] | −63.2 | 63.2 | −60.4 | 59.8 | 60.9 | −59.8 | 49.2 | 70.1 | 64.3 | 58.9 |
| A-centroid–B-centroid distance $d_{AB}$ [Å] | 4.867 | 4.867 | 4.857 | 4.863 | 4.889 | 4.866 | 4.790 | 4.965 | 4.963 | 4.973 |
| 2) Side chain at N5/C5 | | | | | | | | | | |
| C13–C5/N5–C16–C17 torsion angle [°] | −142.5 | −71.2 | −142.3 | −69.5 | −70.4 | −141.3 | −159.7 | −68.5 | 179.1 | −174.3 |
| C5/N5–C16–C17–C18 torsion angle [°] | 71.1 | −71.2 | −177.8 | 178.1 | 177.1 | −176.5 | 160.5 | −179.6 | –113.0 | 121.2 |
| N5'–A-ring centroid distance $d_{NA}$ [Å] | 6.553 | 6.107 | 6.197 | 6.541 | 6.533 | 6.176 | 6.537 | 6.248 | 7.330 | 7.301 |
| N5'–B-ring centroid distance $d_{NB}$ [Å] | 6.107 | 6.553 | 6.495 | 6.106 | 6.088 | 6.488 | 6.076 | 7.219 | 5.955 | 5.765 |

(b) Complex with proteins

| CSD/PDB code [d] | 2Q6H [l] | | 2QEI [m] | | 4MMA [n] | 6G9I [p] | 3RZE [q] | |
|---|---|---|---|---|---|---|---|---|
| | CPM | | CPM | | CPM | CPM | DXP | |
| | 7-Cl | 3-Cl | 7-Cl | 3-Cl | 3-Cl | 3-Cl | *E* form | *Z* form |
| 1) [6-7-6]-Tricyclic core | | | | | | | | |
| Butterfly angle [°] [a] | 128.6 | 120.7 | 126.4 | 120.7 | 136.0 | 130.2 | 123.3 | 123.7 |
| Annellation angle [°] [b] | 28.9 | 30.6 | 29.1 | 30.6 | 30.6 | 32.5 | 24.4 | 25.8 |
| Twist angle [°] [c] | 10.6 | 7.4 | 9.6 | 7.4 | −13.5 | 15.5 | −10.7 | 13.5 |
| C15–C10–C11–C12 torsion angle [°] | 54.3 | 42.0 | 47.4 | 42.0 | −76.9 | 76.8 | −66.1 | 69.3 |
| A-centroid–B-centroid distance $d_{AB}$ [Å] | 5.037 | 4.881 | 4.988 | 4.881 | 5.037 | 4.947 | 4.910 | 4.923 |
| 2) Side chain at N5/C5 | | | | | | | | |
| C13–C5/N5–C16–C17 torsion angle [°] | 179.1 | −131.2 | −160.4 | −131.2 | −90.0 | −135.2 | 1.0 | −179.4 |
| C5/N5–C16–C17–C18 torsion angle [°] | 92.9 | 85.2 | 59.6 | 85.2 | 167.7 | −145.0 | 112.8 | 99.11 |
| N5'–A-ring centroid distance $d_{NA}$ [Å] | 7.446 | 5.927 | 7.150 | 5.927 | 6.207 | 7.274 | 6.256 | 6.997 |
| N5'–B-ring centroid distance $d_{NB}$ [Å] | 5.303 | 5.406 | 5.408 | 5.406 | 5.281 | 5.577 | 6.918 | 6.089 |

**Table S6.** Continued.

(b) Complex with proteins

| CSD/PDB code [d] | 2QB4 [r] | | 2QJU [s] | 2Q72 [t] | | 6G9B [u] | | 4M48 [v] | 3APV [w] | |
|---|---|---|---|---|---|---|---|---|---|---|
| | DPM | | DPM | IPM | | IPM | | NRT | AMT | |
| | Mol 1 | Mol 2 | | Mol 1 | Mol 2 | Mol 1 | Mol 2 | | Mol 1 | Mol 2 |
| 1) [6-7-6]-Tricyclic core | | | | | | | | | | |
| Butterfly angle [°] [a] | 127.5 | 129.3 | 120.5 | 129.2 | 126.0 | 111.6 | 110.1 | 124.2 | 122.8 | 120.5 |
| Annellation angle [°] [b] | 28.3 | 28.5 | 29.2 | 29.1 | 29.0 | 32.4 | 33.5 | 29.1 | 27.6 | 27.7 |
| Twist angle [°] [c] | 10.8 | −8.7 | 9.7 | −9.2 | 11.2 | −14.4 | −12.1 | 14.7 | 9.6 | 11.8 |
| C15–C10–C11–C12 torsion angle [°] | 57.9 | −50.8 | 54.5 | −48.9 | 55.8 | −62.8 | −61.5 | 72.0 | 59.8 | 62.9 |
| A-centroid–B-centroid distance $d_{AB}$ [Å] | 5.016 | 5.036 | 4.855 | 5.021 | 5.101 | 4.781 | 4.757 | 4.950 | 4.920 | 4.878 |
| 2) Side chain at N5/C5 | | | | | | | | | | |
| C13–C5/N5–C16–C17 torsion angle [°] | −168.1 | 68.7 | 179.5 | 73.0 | −167.6 | −144.7 | −138.3 | –0.1 | −178.8 | 179.2 |
| C5/N5–C16–C17–C18 torsion angle [°] | 77.0 | 89.4 | 81.4 | 85.8 | 69.8 | 165.2 | 145.9 | 162.1 | 128.7 | 128.7 |
| N5'–A-ring centroid distance $d_{NA}$ [Å] | 7.331 | 6.697 | 7.767 | 6.722 | 7.293 | 7.297 | 7.012 | 6.571 | 6.152 | 6.188 |
| N5'–B-ring centroid distance $d_{NB}$ [Å] | 5.373 | 6.014 | 5.170 | 5.949 | 5.430 | 6.508 | 6.714 | 6.307 | 5.153 | 5.224 |

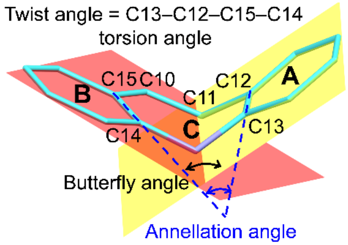


[a,b,c] Angle between aromatic planes A and B; Angle between C13–C12 and C14–C15; C13–C12–C15–C14 torsion angle (see inset for atom numbering).

[d] For the β-CD encapsulation of DPM, IPM [13] and NRT, AMT [18], see Table 1 and Figure 3.

[e,f,g,h,i] CSD codes: CIMPRA [24]; PUKGEI [25]; IMIPRC [26]; JINGIW [27]; YOVZEO [28]; none of free DXP HCl or DXP in complex with small molecule has been reported.

[j] Two CPM molecules are inversion-symmetry-related, i.e., crystals belong to monoclinic, space group $P2_1/c$.

[k] Number of molecules in the asymmetric unit.

[l] CPM in complex with bacterial leucine transporter (LeuT), L-leucine and sodium (PDB code: 2Q6H) [29].

[m] CPM in complex with LeuT, L-alanine and sodium (PDB code: 2QEI) [29].

[n] CPM in complex with biogenic leucine transporter (LeuBAT) (PDB code: 4MMA) [30].

[p] CPM in complex with ebolavirus glycoprotein (PDB code: 6G9I) [31].

[q] DXP in complex with human histamine $H_1$ (PDB code: 3RZE) [32].

[r] DPM in complex with LeuT, L-leucine and sodium (PDB code: 2QB4) [29].

[s] DPM in complex with LeuT and L-leucine (PDB code: 2QJU) [33].

[t] IPM in complex with LeuT, L-leucine and sodium (PDB code 2Q72) [29].

[u] IPM in complex with ebolavirus glycoprotein (PDB code: 6G9B) [31].

[v] NRT in complex with dopamine transporter(DAT), NaCl and cholesterol (PDB code: 4M48) [34].

[w] AMT in complex with human alpha1-acid glycoprotein (PDB code: 3APV) [35].

## 3. Computational data

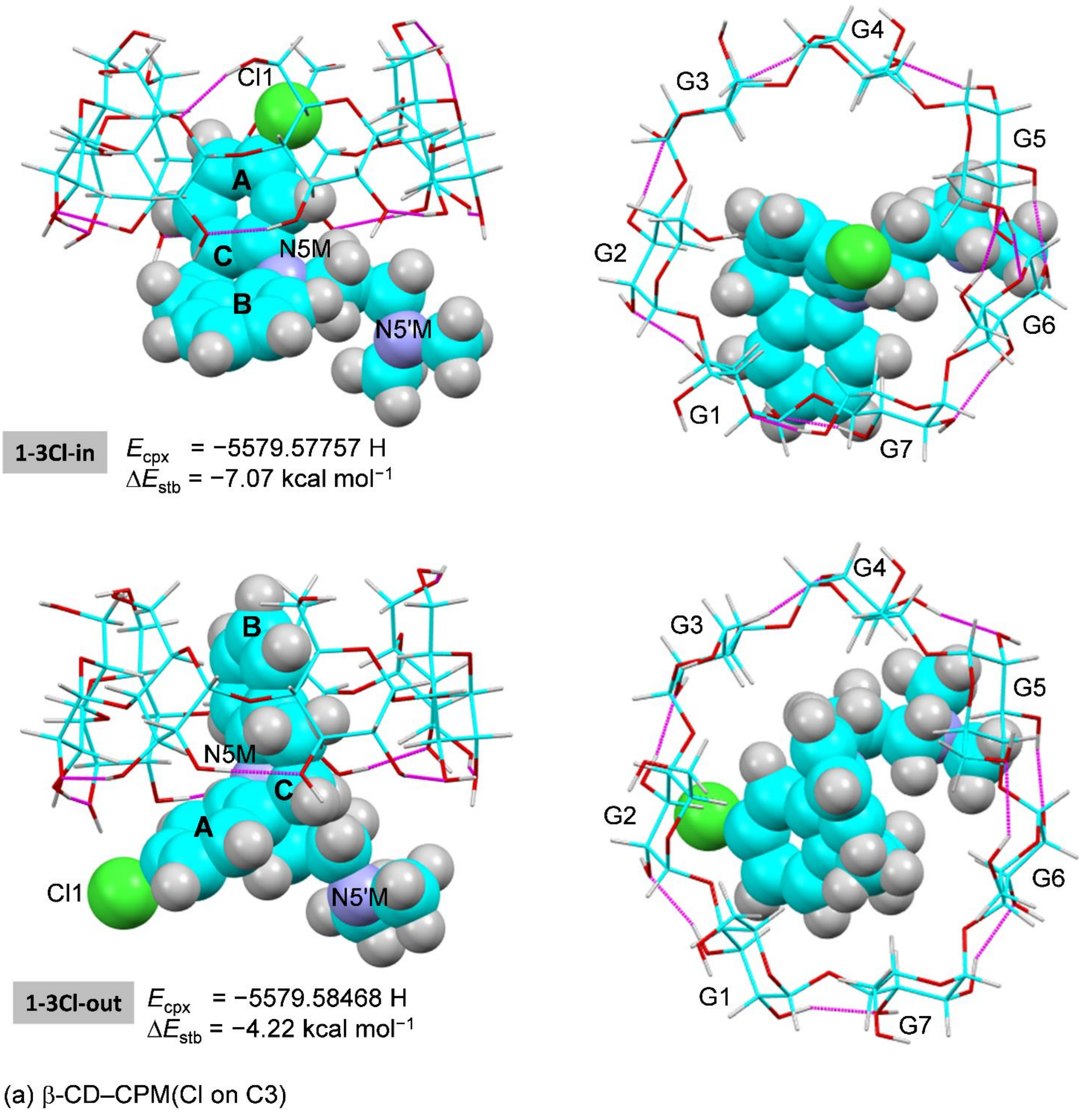


**Figure S1.** Inclusion complexes of (a) β-CD−CPM(Cl on C3), (b) β-CD−CPM(Cl on C7) with Cl inside and outside the β-CD cavity, and (c) β-CD−*E*/*Z*-DXP, derived from DFT complete-geometry optimization in the gas phase; side view (left) and top view (right). For better comparison, the energy of complex ($E_{cpx}$) and stabilization energy ($\Delta E_{stb}$) are given; see also Tables S7 and 3. The H-bonding interactions are indicated by magenta lines.

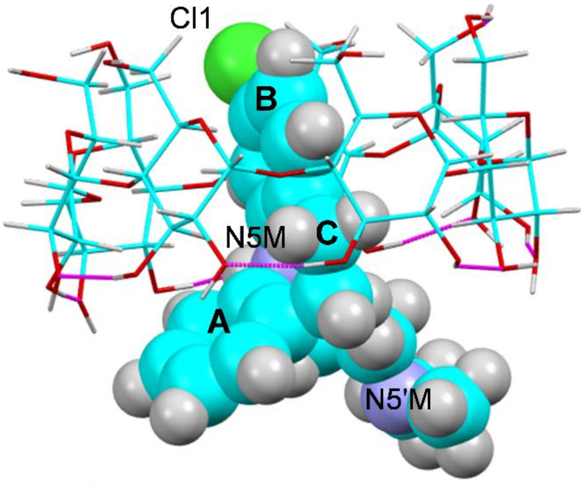


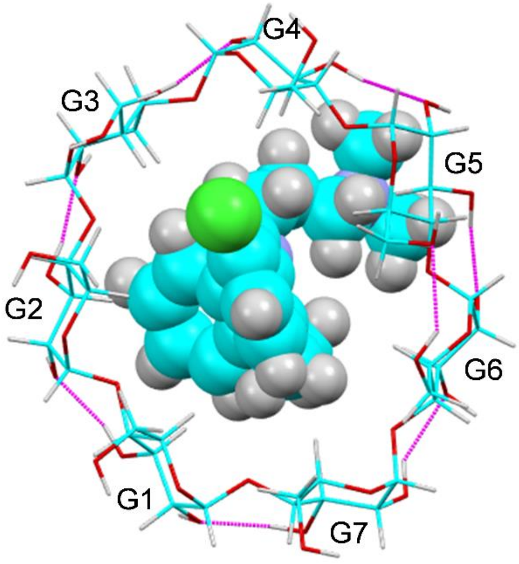


**1-7Cl-in** $E_{\text{cpx}}$ = −5579.59119 H
$\Delta E_{\text{stb}}$ = −5.17 kcal mol$^{-1}$

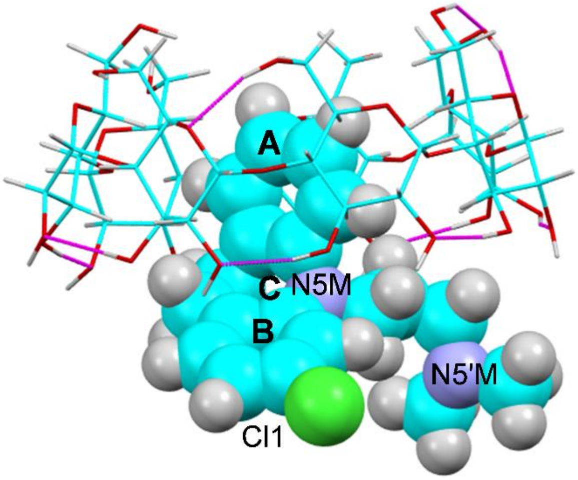


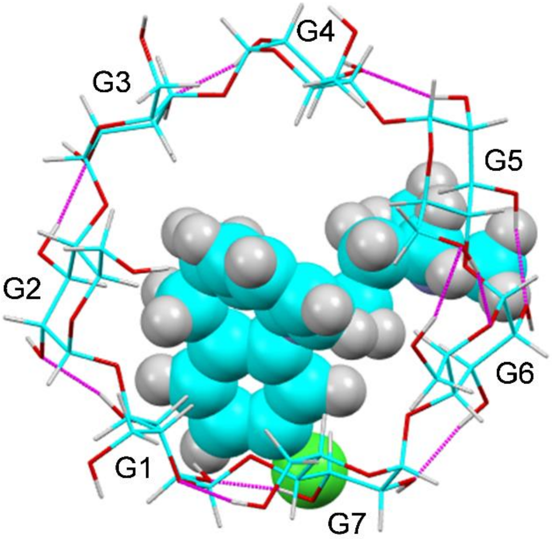


**1-7Cl-out** $E_{\text{cpx}}$ = −5579.57582 H
$\Delta E_{\text{stb}}$ = −5.62 kcal mol$^{-1}$

(b) β-CD–CPM(Cl on C7)

**Figure S1.** Continued.

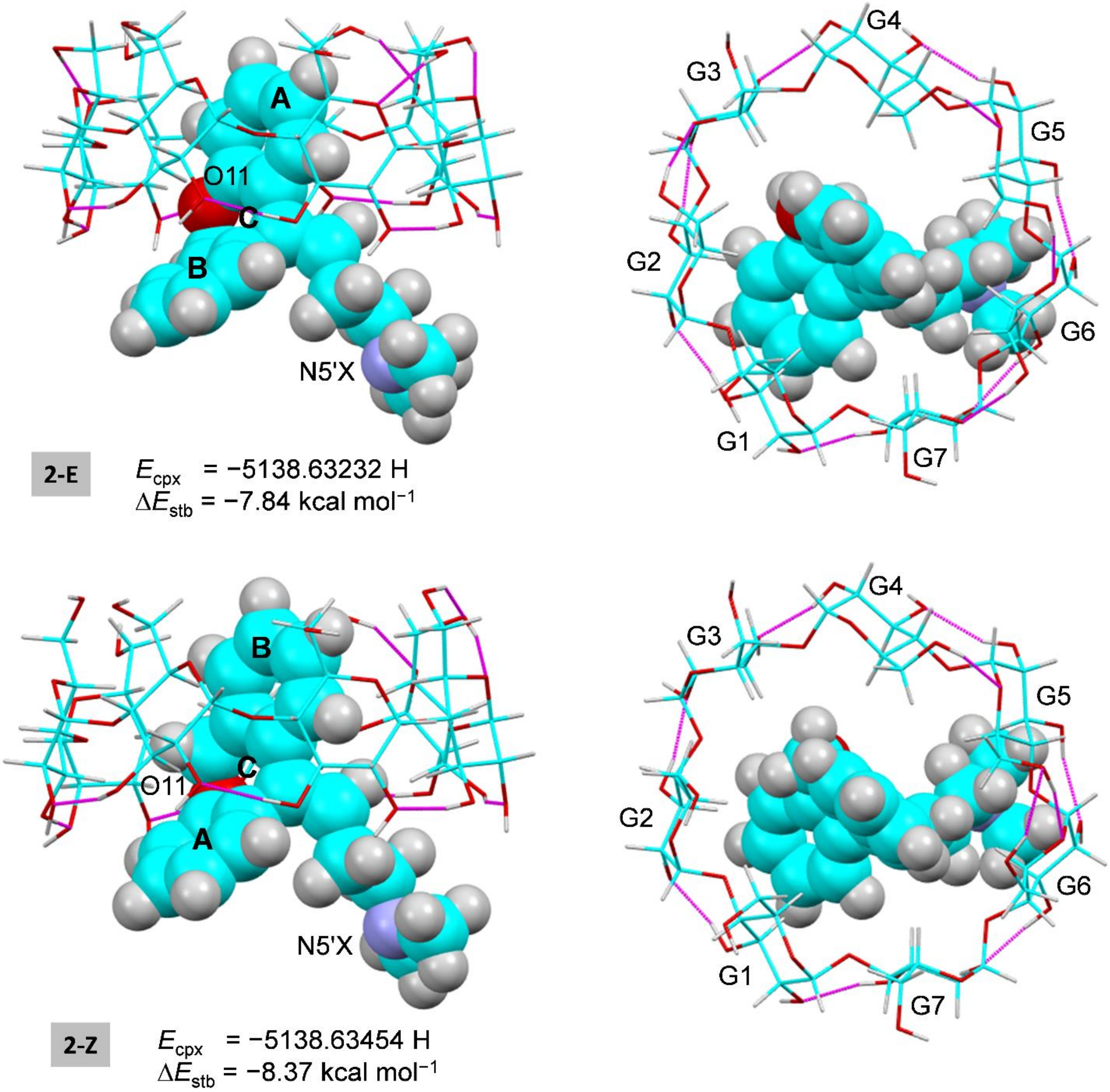


(c) β-CD–DXP

**Figure S1.** Continued.

**Table S7.** Hydrogen bond parameters in β-CD–CPM (4 modes) and β-CD–DXP (2 modes) inclusion complexes from DFT full-geometry optimization [Å, º]. [a]

| D–H···A | D–H | H···A | D···A | ∠(DHA) | D–H···A | D–H | H···A | D···A | ∠(DHA) |
|---|---|---|---|---|---|---|---|---|---|
| β-CD·CMP [b] | | | | | | | | | |
| **1-3Cl-in** | | | β–CD | conformation | | | | | |
| β-*CD*–β-*CD* | | | Distorted | round | β-*CD*–*CMP* | | | | |
| O31–H···O22 | 0.98 | 1.95 | 2.91 | 167.4 | C31–H···*Cg*1 [c] | 1.10 | 3.55 | 4.62 | 163.7 |
| O32–H···O23 | 0.98 | 1.91 | 2.87 | 167.3 | O21–H···*Cg*2 [c] | 0.98 | 2.84 | 3.47 | 123.1 |
| O24–H···O33 | 0.98 | 1.99 | 2.93 | 162.4 | | | | | |
| O25–H···O34 | 0.98 | 2.12 | 3.05 | 158.8 | | | | | |
| O35–H···O26 | 0.98 | 2.12 | 3.07 | 165.3 | | | | | |
| O65–H···O56 | 0.98 | 1.88 | 2.85 | 166.5 | | | | | |
| O36–H···O27 | 0.98 | 1.93 | 2.90 | 168.2 | | | | | |
| O66–H···O65 | 0.97 | 2.28 | 3.12 | 143.4 | | | | | |
| O37–H···O21 | 0.98 | 1.92 | 2.88 | 164.6 | | | | | |
| O67–H···O51 | 0.98 | 2.06 | 3.01 | 163.7 | | | | | |
| **1-3Cl-out** | | | | | | | | | |
| β-*CD*–β-*CD* | | | Distorted | round | β-*CD*–*CMP* | | | | |
| O21–H···O37 | 0.98 | 2.07 | 2.99 | 157.4 | C52–H···*Cg*2 | 1.10 | 3.46 | 4.50 | 157.7 |
| O31–H···O22 | 0.98 | 1.98 | 2.93 | 164.6 | C55–H···*Cg*2 | 1.10 | 3.36 | 4.36 | 151.6 |
| O32–H···O23 | 0.98 | 1.96 | 2.92 | 164.1 | | | | | |
| O33–H···O24 | 0.98 | 1.96 | 2.92 | 168.2 | | | | | |
| O34–H···O25 | 0.98 | 1.94 | 2.91 | 166.8 | | | | | |
| O35–H···O26 | 0.98 | 1.98 | 2.93 | 163.4 | | | | | |
| O66–H···O65 | 0.98 | 2.29 | 3.11 | 140.9 | | | | | |
| O27–H···O36 | 0.98 | 2.05 | 2.96 | 153.7 | | | | | |
| **1-7Cl-in** | | | | | | | | | |
| β-*CD*–β-*CD* | | | Distorted | round | β-*CD*–*CMP* | | | | |
| O31–H···O22 | 0.98 | 1.91 | 2.86 | 164.6 | C52–H···*Cg*2 | 1.10 | 3.59 | 4.67 | 167.5 |
| O32–H···O23 | 0.98 | 1.94 | 2.91 | 166.7 | C55–H···*Cg*2 | 1.10 | 3.48 | 4.43 | 145.6 |
| O33–H···O24 | 0.98 | 1.95 | 2.92 | 168.3 | C32–H···*Cg*1 | 1.10 | 3.57 | 4.55 | 150.2 |
| O34–H···O25 | 0.98 | 1.94 | 2.91 | 168.0 | | | | | |
| O35–H···O26 | 0.98 | 1.96 | 2.91 | 164.0 | | | | | |
| O66–H···O65 | 0.97 | 2.41 | 3.22 | 140.0 | | | | | |
| O27–H···O36 | 0.98 | 2.18 | 3.02 | 143.6 | | | | | |
| O37–H···O21 | 0.98 | 1.94 | 2.91 | 170.2 | | | | | |
| **1-7Cl-out** | | | | | | | | | |
| β-*CD*–β-*CD* | | | Distorted | round | β-*CD*–*CMP* | | | | |
| O31–H···O22 | 0.98 | 1.93 | 2.90 | 168.2 | O21–H···*Cg*2 | 0.98 | 3.11 | 3.69 | 119.9 |
| O32–H···O23 | 0.98 | 1.91 | 2.87 | 168.2 | | | | | |
| O24–H···O33 | 0.98 | 1.98 | 2.93 | 162.6 | | | | | |
| O25–H···O34 | 0.98 | 2.13 | 3.07 | 160.1 | | | | | |
| O35–H···O26 | 0.98 | 2.14 | 3.10 | 164.4 | | | | | |
| O65–H···O56 | 0.98 | 1.89 | 2.85 | 164.9 | | | | | |
| O36–H···O27 | 0.98 | 1.92 | 2.89 | 168.6 | | | | | |
| O66–H···O65 | 0.97 | 2.19 | 3.05 | 146.2 | | | | | |
| O37–H···O21 | 0.98 | 1.93 | 2.88 | 164.7 | | | | | |
| O67–H···O51 | 0.98 | 2.04 | 2.99 | 162.5 | | | | | |

**Table S7.** Continued.

| D–H···A | D–H | H···A | D···A | ∠(DHA) | D–H···A | D–H | H···A | D···A | ∠(DHA) |
|---|---|---|---|---|---|---|---|---|---|
| β-CD·DXP [b] | | | | | | | | | |
| **2-E** | | | | | | | | | |
| β-*CD*–β-*CD* | | | Distorted | round | β-*CD*–*E*-*DXP* | | | | |
| O31–H···O22 | 0.98 | 1.88 | 2.84 | 167.1 | C31–H···*Cg*2 | 1.10 | 3.75 | 4.62 | 137.8 |
| O32–H···O23 | 0.98 | 2.04 | 2.98 | 161.0 | | | | | |
| O62–H···O53 | 0.98 | 2.02 | 2.96 | 159.7 | | | | | |
| O24–H···O33 | 0.98 | 2.01 | 2.97 | 164.1 | | | | | |
| O64–H···O55 | 0.98 | 1.97 | 2.88 | 155.4 | | | | | |
| O25–H···O34 | 0.98 | 2.11 | 3.03 | 156.9 | | | | | |
| O35–H···O26 | 0.98 | 2.03 | 2.99 | 164.8 | | | | | |
| O65–H···O56 | 0.98 | 2.00 | 2.93 | 158.3 | | | | | |
| O36–H···O27 | 0.98 | 1.97 | 2.94 | 166.8 | | | | | |
| O66–H···O57 | 0.98 | 2.04 | 2.98 | 161.6 | | | | | |
| O37–H···O21 | 0.98 | 1.88 | 2.86 | 168.9 | | | | | |
| **2-Z** | | | | | | | | | |
| β-*CD*–β-*CD* | | | Distorted | round | β-*CD*–*Z*-*DXP* | | | | |
| O31–H···O22 | 0.98 | 1.90 | 2.86 | 164.8 | C51–H···*Cg*2 | 1.10 | 3.41 | 4.46 | 167.5 |
| O32–H···O23 | 0.98 | 1.98 | 2.95 | 167.2 | C55–H···*Cg*2 | 1.10 | 3.64 | 4.57 | 145.6 |
| O24–H···O33 | 0.98 | 2.02 | 2.97 | 162.6 | C31–H···*Cg*1 | 1.10 | 3.50 | 4.62 | 137.8 |
| O64–H···O55 | 0.98 | 1.97 | 2.89 | 155.0 | C32–H···*Cg*1 | 1.10 | 3.62 | 4.55 | 150.2 |
| O25–H···O34 | 0.98 | 2.07 | 2.99 | 156.8 | | | | | |
| O35–H···O26 | 0.98 | 2.07 | 3.02 | 163.7 | | | | | |
| O65–H···O56 | 0.98 | 1.91 | 2.87 | 167.2 | | | | | |
| O36–H···O27 | 0.98 | 1.88 | 2.85 | 168.7 | | | | | |
| O66–H···O65 | 0.97 | 2.37 | 3.21 | 143.2 | | | | | |
| O37–H···O21 | 0.98 | 1.92 | 2.89 | 167.1 | | | | | |

[a] DFT energy minimization in vacuum at the B3LYP/6–31+G(d)/4–31G level, see also Figure S1 and Table 3.
[b] X-ray-derived structures are used as starting models.
[c] C/O–H···π interactions with *Cg*1 and *Cg2* as the centroids of A-ring (C1–C2–C3–C4–C13–C12) and B-ring (C6–C7–C8–C9–C15–C14), respectively.

## References


1. Misiuk, W.; Zalewska, M. Study on the inclusion interactions of β-cyclodextrin and its derivative with clomipramine by spectroscopy and its analytic application. *Anal. Lett.* **2008**, *41*, 543–560. https://doi.org/10.1080/00032710801910650.
2. Georgiou, M. E.; Koupparis, M. A.; Georgiou, C. A. Rapid automated spectrophotometric competitive complexation studies of drugs with cyclodextrins using the flow injection gradient technique: tricyclic antidepressant drugs with α-cyclodextrin. *Analyst* **1999**, *124*, 391–396. https://doi.org/10.1039/A808426B.
3. Rajendiran, N.; Sankaranarayanan, R. K.; Saravanan, J. A study of supramolecular host–guest interaction of dothiepin and doxepin drugs with cyclodextrin macrocycles. *J. Mol. Struct.* **2014**, *1067*, 252–260. https://doi.org/10.1016/j.molstruc.2014.03.051.
4. Valsami, G. N.; Koupparis, M. A.; Macheras, P. E. Complexation studies of cyclodextrins with tricyclic antidepressants using ion-selective electrodes. *Pharm. Res.* **1992**, *9*, 94–100. https://doi.org/10.1023/A:1018940013006.
5. Cruz, J. R.; Becker, B. A.; Morris, K. F.; Larive, C. K. NMR characterization of the host–guest inclusion complex between β-cyclodextrin and doxepin. *Magn. Reson. Chem*. **2008**, *46*, 838–845. https://doi.org/10.1002/mrc.2267.
6. Sankaranarayanan, R. K.; Siva, S.; Venkatesh, G.; Prabhu, A. A. M.; Rajendiran, N. Dual fluorescence of dothiepin, doxepin drugs—effect of solvents and β-cyclodextrin. *J. Mol. Liq.* **2011**, *161*, 107–114. https://doi.org/10.1016/j.molliq.2011.04.016.
7. Jones, C. J.; Larive, C. K. Microcoil NMR study of the interactions between doxepin, β-cyclodextrin, and acetate during capillary isotachophoresis. *Anal. Chem.* **2012**, *84*, 7099–7106. https://doi.org/10.1021/ac301401p.
8. Jenita, M. J.; Prabhu, A.; Rajendiran, N. Theoretical study of inclusion complexation of tricyclic antidepressant drugs with β-cyclodextrin. *Ind. J. Chem.* **2012**, *51A*, 1686–1694. http://nopr.niscair.res.in/handle/123456789/15201.
9. Junquera, E.; Romero, J. C.; Aicart, E. Behavior of tricyclic antidepressants in aqueous solution: self-aggregation and association with β-cyclodextrin. *Langmuir* **2001**, *17*, 1826–1832. https://doi.org/10.1021/la000819q.
10. Jalali, F.; Ezzati, N. Spectrofluorimetric study and determination of desipramine in the presence of β-cyclodextrin. *J. Anal. Chem.* **2014**, *69*, 367–370. https://doi.org/10.1134/S1061934814040029.
11. Misiuk, W. Study on the Inclusion Complex Formation of Desipramine with Β-Cyclodextrin and Its Pharmaceutical Application. *W. J. Pharm. Pharm. Sci.* **2015**, *4*, 18–33. https://www.wjpps.com/Wjpps_controller/abstract_id/3840.
12. Viswalingam, M.; Prabu, S.; Sivakumar, K.; Rajamohan, R. Spectral characteristics of desipramine in β-cyclodextrin cavity through inclusion complex. *J. Macromol. Sci.* **2016**, *A53*, 781–790. https://doi.org/10.1080/10601325.2016.1237817.
13. Aree, T. β-Cyclodextrin inclusion complexation with tricyclic antidepressants desipramine and imipramine: a structural chemistry perspective. *J. Pharm. Sci.* **2020**, *109*, 3086–3094. https://doi.org/10.1016/j.xphs.2020.07.007.
14. Cano, J.; Rodriguez, A.; Aicart, E.; Junquera, E. Temperature effect on the complex formation between tricyclic antidepressant drugs (amitriptyline or imipramine) and hydroxypropyl-β-cyclodextrin in water. *J. Incl. Phenom. Macrocycl. Chem.* **2007**, *59*, 279–285. https://doi.org/10.1007/s10847-007-9328-x.
15. Sousa, F. B. D.; Denadai, A. M. L.; Lula, I. S.; Nascimento Jr, C. S.; Neto, N. S. F.; Lima, A. C.; ... Sinisterra, R. D. Supramolecular self-assembly of cyclodextrin and higher water soluble guest: Thermodynamics and topological studies. *J. Am. Chem. Soc.* **2008**, *130*, 8426–8436. https://doi.org/10.1021/ja801080v.
16. Viswalingam, M.; Prabu, S.; Sivakumar, K.; Rajamohan, R. Preparation and characterization of a imipramine–β-cyclodextrin inclusion complex. *Instrum. Sci. Technol.* **2016**, *44*, 651–671. https://doi.org/10.1080/10739149.2016.1177726.
17. Kundu, M.; Roy, M. N. Preparation, interaction and spectroscopic characterization of inclusion complex of a cyclic oligosaccharide with an antidepressant drug. *J. Incl. Phenom. Macrocycl. Chem.* **2017**, *89*, 177–187. https://doi.org/10.1007/s10847-017-0745-1.

18. Aree, T. β-Cyclodextrin encapsulation of nortriptyline HCl and amitriptyline HCl: molecular insights from single-crystal X-ray diffraction and DFT calculation. *Int. J. Pharm.* **2020**, *575*, 118899. https://doi.org/10.1016/j.ijpharm.2019.118899.
19. Castiglione, F.; Ganazzoli, F.; Malpezzi, L.; Mele, A.; Panzeri, W.; Raffaini, G. Inclusion complexes of β-cyclodextrin with tricyclic drugs: an X-ray diffraction, NMR and molecular dynamics study. *Beilstein J. Org. Chem.* **2017**, *13*, 714–719. https://doi.org/10.3762/bjoc.13.70.
20. Cremer, D. T.; Pople, J. A. General definition of ring puckering coordinates. *J. Am. Chem. Soc.* **1975**, *97*, 1354-1358. https://doi.org/10.1021/ja00839a011.
21. Aree, T.; Jongrungruangchok, S. Crystallographic evidence for β-cyclodextrin inclusion complexation facilitating the improvement of antioxidant activity of tea (+)-catechin and (−)-epicatechin. *Carbohydr. Polym.* **2016**, *140*, 362–373. https://doi.org/10.1016/j.carbpol.2015.12.066.
22. Lindner, K.; Saenger, W. Crystal and molecular structure of cyclohepta-amylose dodecahydrate. *Carbohydr. Res.* **1982**, *99*, 103–115. https://doi.org/10.1016/S0008-6215(00)81901-1.
23. French, A. D.; Johnson, G. P. Linkage and pyranosyl ring twisting in cyclodextrins. *Carbohydr. Res.* **2007**, 342, 1223–1237. https://doi.org/10.1016/j.carres.2007.02.033.
24. Post, M. L.; Horn, A. S. The crystal and molecular structure of the tricyclic antidepressant chlorimipramine hydrochloride: 3-chloro-5-(3-dimethylaminopropyl)-10,11-dihydro-5H-dibenz[b,f] azepine hydrochloride. *Acta Cryst.* **1977**, *B33*, 2590–2595. https://doi.org/10.1107/S0567740877008966.
25. Jasinski, J. P.; Butcher, R. J.; Hakim Al-Arique, Q. N. M.; Yathirajan, H. S.; Ramesha, A. R. Desipramine hydrochloride: a non-merohedrally twinned structure. *Acta Cryst.* **2010**, *E66*, o674–o675. https://doi.org/10.1107/S1600536810006203.
26. Post, M. L.; Kennard, O.; Horn, A. S. The tricyclic antidepressants: imipramine hydrochloride. The crystal and molecular structure of 5-(3-dimethylaminopropyl)-10,1l-dihydro-5H-dibenz[b,f]azepine hydrochloride. *Acta Cryst.* **1975**, *B31*, 1008–1013. https://doi.org/10.1107/S0567740875004396.
27. Klein, C. L.; Banks, T. A.; Rouselle, D. Structure of nortriptyline hydrochloride. *Acta Cryst.* **1991**, *C47*, 1478–1480. https://doi.org/10.1107/S010827019001280X.
28. Klein, C. L.; Lear, J.; O'Rourke, S.; Williams, S.; Liang, L. Crystal and molecular structures of tricyclic neuroleptics. *J. Pharm. Sci.* **1994**, *83*, 1253–1256. https://doi.org/10.1002/jps.2600830914.
29. Singh, S. K.; Yamashita, A.; Gouaux, E. Antidepressant binding site in a bacterial homologue of neurotransmitter transporters. *Nature* **2007**, *448*, 952–956. https://doi.org/10.1038/nature06038.
30. Wang, H.; Goehring, A.; Wang, K. H.; Penmatsa, A.; Ressler, R.; Gouaux, E. Structural basis for action by diverse antidepressants on biogenic amine transporters. *Nature* **2013**, *503*, 141–146. https://doi.org/10.1038/nature12648.
31. Zhao, Y.; Ren, J.; Fry, E. E.; Xiao, J.; Townsend, A. R.; Stuart, D. I. Structures of ebola virus glycoprotein complexes with tricyclic antidepressant and antipsychotic drugs. *J. Med. Chem.* **2018**, *61*, 4938–4945. https://doi.org/10.1021/acs.jmedchem.8b00350.
32. Shimamura, T.; Shiroishi, M.; Weyand, S.; Tsujimoto, H.; Winter, G.; Katritch, V.; ... Iwata, S. Structure of the human histamine $H_1$ receptor complex with doxepin. *Nature* **2011**, *475*, 65–70. https://doi.org/10.1038/nature10236.
33. Zhou, Z.; Zhen, J.; Karpowich, N. K.; Goetz, R. M.; Law, C. J.; Reith, M. E.; Wang, D. N. LeuT-desipramine structure reveals how antidepressants block neurotransmitter reuptake. *Science* **2007**, *317*, 1390–1393. https://doi.org/10.1126/science.1147614.
34. Penmatsa, A.; Wang, K. H.; Gouaux, E. X-ray structure of dopamine transporter elucidates antidepressant mechanism. *Nature* **2013**, *503*, 85–90. https://doi.org/10.1038/nature12533.
35. Nishi, K.; Ono, T.; Nakamura, T.; Fukunaga, N.; Izumi, M.; Watanabe, H.; ... Otagiri, M. Structural insights into differences in drug-binding selectivity between two forms of human $\alpha$1-acid glycoprotein genetic variants, the A and F1* S forms. *J. Biol. Chem.* **2011**, *286*, 14427–14434. https://doi.org/10.1074/jbc.M110.208926.
